# Highly contaminated river otters (*Lontra canadensis*) are effective biomonitors of environmental pollutant exposure

**DOI:** 10.1007/s10661-022-10272-9

**Published:** 2022-08-16

**Authors:** Michelle Wainstein, Louisa B. Harding, Sandra M. O’Neill, Daryle T. Boyd, Fred Koontz, Bobbi Miller, Cornelya F. C. Klütsch, Philippe J. Thomas, Gina M. Ylitalo

**Affiliations:** 1 Conservation, Research and Education Opportunities, Seattle, WA 98107 USA; 2 Washington Department of Fish and Wildlife, PO Box 43200, Olympia, WA 98504-3200 USA; 3 Northwest Fisheries Science Center, National Marine Fisheries Service, National Oceanic and Atmospheric Administration, 2725 Montlake Boulevard East, Seattle, WA 98112-2097 USA; 4 Woodland Park Zoo, 5500 Phinney Ave N, Seattle, WA 98103 USA; 5 Division of Environment and Natural Resources, Norwegian Institute of Bioeconomy Research (NIBIO), NIBIO Svanhovd, NO-9925 Svanvik, Norway; 6 Science and Technology Branch, Environment and Climate Change Canada, National Wildlife Research Center, 1125 Colonel By Drive, Raven Road, Ottawa, ON K1A 0H3 Canada

**Keywords:** River otter, Persistent organic pollutants, Polychlorinated biphenyls (PCBs), Polycyclic aromatic hydrocarbons (PAHs), Biomonitor, Superfund site

## Abstract

River otters (*Lontra canadensis*) are apex predators that bioaccumulate contaminants via their diet, potentially serving as biomonitors of watershed health. They reside throughout the Green-Duwamish River, WA (USA), a watershed encompassing an extreme urbanization gradient, including a US Superfund site slated for a 17-year remediation. The objectives of this study were to document baseline contaminant levels in river otters, assess otters’ utility as top trophic-level biomonitors of contaminant exposure, and evaluate the potential for health impacts on this species. We measured a suite of contaminants of concern, lipid content, nitrogen stable isotopes (δ^15^N), and microsatellite DNA markers in 69 otter scat samples collected from twelve sites. Landcover characteristics were used to group sampling sites into industrial (Superfund site), suburban, and rural development zones. Concentrations of polychlorinated biphenyls (PCBs), polybrominated diphenyl ether flame-retardants (PBDEs), dichlorodiphenyl-trichloroethane and its metabolites (DDTs), and polycyclic aromatic hydrocarbons (PAHs) increased significantly with increasing urbanization, and were best predicted by models that included development zone, suggesting that river otters are effective biomonitors, as defined in this study. Diet also played an important role, with lipid content, δ^15^N or both included in all best models. We recommend river otter scat be included in evaluating restoration efforts in this Superfund site, and as a potentially useful monitoring tool wherever otters are found. We also report ΣPCB and ΣPAH exposures among the highest published for wild river otters, with almost 70% of samples in the Superfund site exceeding established levels of concern.

**Supplementary information:**

The online version contains supplementary material available at 10.1007/s10661-022-10272-9.

## Introduction

Anthropogenically derived environmental contaminants are now globally ubiquitous and pose an urgent concern for environmental, wildlife, and human health (Ashraf, 2017; Dietz et al., 2019). Persistent organic pollutants (POPs) are a particularly insidious class of compounds because of their namesake characteristics: they are highly resistant to degradation, readily bioavailable and toxic across many species, including humans (Johnson et al., 2013; Jones & de Voogt, 1999). Furthermore, POPs disperse easily throughout the environment from local and long-range sources and are lipophilic, resulting in bioaccumulation in the lipid-rich tissues of biota and biomagnification in food webs (Elliott et al., 2009; Fremlin et al., 2020; Giesy & Kannan, 1998; Gobas & Arnot, 2010). POPs include a wide variety of toxic compounds, including polychlorinated biphenyls (PCBs), polybrominated diphenyl ether flame-retardants (PBDEs), chlorinated pesticides such as dichlorodiphenyl-trichloroethane and its metabolites (DDTs), chlordanes (CHLDs), and hexachlorobenzene (HCB). Although international and national regulatory frameworks have decreased production and open use of some classes of POPs, they are by definition persistent, and new classes of POPs regularly emerge.

Polycyclic aromatic hydrocarbons (PAHs) are another class of organic pollutants that frequently co-occur with POPs in urban and industrial regions. They include hydrocarbons derived from petroleum production, oils seeps, combustion of wood and fossil fuels, as well as biogenically formed PAHs (Collier et al., 2014). Global industrialization has driven an increase in the amounts and rates of their introduction and mobilization into the environment (Farrington & Takada, 2014). Various PAHs exhibit similar properties to POPs: they are omnipresent and persistent, they accumulate in sediments and in the tissues of invertebrates, and are highly toxic to numerous species (see Behera et al., 2018; Collier et al., 2014). However, unlike POPs, these compounds have been shown to be rapidly metabolized and therefore found in low concentration in various species of vertebrates (Meador et al., 1995; Varanasi et al., 1989; Ylitalo et al., 2017), though resulting secondary metabolites can be toxic as well (e.g., Lee et al., 2017). Because of low water solubility, both POPs and PAHs preferentially bind to sedimentary organic carbon in aquatic systems, creating large legacy reservoirs in sediments in shallow water systems with the potential for continuous reintroduction into local aquatic food web dynamics (Long et al., 2005; Malins et al., 1984).

River otter species are apex predators in aquatic ecosystems (Kruuk, 2006; Lariviere & Walton, 1998; Larsen, 1984) that can have relatively localized and seasonally constant home and core ranges (e.g., Bowyer et al., 1995; Gorman et al., 2006; Helon et al., 2004; Sauer et al., 1999). They are vulnerable to contaminant exposure through their diet and biomagnification of environmental pollutants (Leonards et al., 1997; Ruus et al., 2002; Smit et al., 1996; Walker, 1990). As such, they have been studied to understand direct impacts on their populations, as potential biomonitors of wildlife exposure to toxics, and as sentinels of environmental contamination and habitat quality (Basu et al., 2007; Carpenter et al., 2014; Hecker et al., 1997; Henny et al., 1981; Mason & Macdonald, 1986; Roos et al., 2001; Taylor et al., 2000). Using a variety of tissue types and, more recently, fecal deposits (scat or spraints), numerous studies have considered chronic exposure to contaminants as a factor in river otter population dynamics in North America and Europe (Henny et al., 1981; Leonards et al., 2008; Mason, 1989; Roos et al., 2001; Wren, 1991). While some have argued a central role for PCBs in dramatic Eurasian otter (*Lutra lutra*) declines (Gutleb, 2002; Leonards et al., 1996; Macdonald & Mason, 1994; Mason, 1996; Roos et al., 2001), others caution that habitat destruction and fragmentation, prey availability, hunting, and exposure to other classes of contaminants should not be ignored as contributing factors (Boyle, 2006; Chanin & Jefferies, 1978; Kruuk, 1997; Kruuk & Conroy, 1996; Melquist et al., 2003; Raesly, 2001). Some of the most extensive studies in North America have been conducted in southern British Columbia, where contaminant loads in North American river otter (*Lontra canadensis*) populations exceeded levels suspected of having adverse effects (Elliott et al., 2008). Further research demonstrated that otters with the highest contaminant concentrations were those utilizing the industrial areas of Victoria and Esquimalt Harbors (Guertin et al., 2010). While pollutants did not appear to have impacts at the population level, hormone concentrations may be affected and conditions suggest elements of an ecological “trap” for otters (Huang et al., 2018).

North American river otters are established residents of the Green-Duwamish River, a major drainage flowing into Puget Sound, a large, multi-basin estuarine ecosystem in Washington State, USA. The river runs a course from the forested Cascade Mountains through regions of protected city water supplies, agriculture, protected green belts, parks, light industry, commercial and residential properties, and heavy industrial use. The Lower Duwamish Waterway (LDW) comprises the final five miles of the Green-Duwamish River as it empties into Seattle’s Elliott Bay (USEPA, 2014); the waterway and adjacent upland areas have served as the city’s major industrial corridor since the river was straightened and dredged in the early 1900s. As early as 1945, the types, quantities, and sources of pollution were documented in the Green-Duwamish River (Foster, 1945). Over the past four decades, studies of the LDW have measured contaminant levels, mapped sources and distributions of sediment contaminants, estimated risks associated with exposure to contaminated sediments, modeled movement and fate of sediments, and evaluated options for cleanup (Conn et al., 2018; Gries & Sloan, 2009; Rodenburg & Leidos, 2017; Sloan & Gries, 2009; USEPA, 2014; Varanasi et al., 1993; Windward, 2007). At least two dozen assessments have been conducted of the biota that reside or migrate through the LDW; most relate either to federally and state-listed salmonid species or impacts of restoration efforts on invertebrate communities (see review in Windward, 2007). Fish and invertebrate samples from the Green-Duwamish River have also been included in regional ecotoxicology research (Lanksbury et al., 2013; O’Neill et al., 2015).

The LDW was designated a United States Environmental Protection Agency (USEPA) Superfund site in 2001 due to high historical industrial pollution, including PCBs and PAHs. As part of remediation plan development, an LDW Baseline Ecological Risk Assessment selected representative “receptors of concern” species from numerous taxa to evaluate existing contaminant loads (Windward, 2007). The North American river otter was chosen (along with the harbor seal, *Phoca vitulina*) to represent mammals in the system. The study determined that PCBs pose a risk for adverse effects to otters in the LDW; however, these conclusions were derived strictly from calculations that estimated exposure based on toxics loads in prey items and assumptions about otter body weights, prey ingestions rates and diet composition (Windward, 2007). There have been no empirical studies of contaminant levels in mammals or apex predators in the LDW or Green-Duwamish River system.

In 2014, after 13 years of research and discussion, the USEPA released its 17-year plan for remediation and restoration of the LDW Superfund site (USEPA, 2014). This presented a unique opportunity to assess conditions prior to the launch of this comprehensive effort to reduce contaminant burdens in this ecological system. Meanwhile, the LDW marks one extreme of the urbanization gradient of the Green-Duwamish River. This system’s parallel gradient in environmental quality presented another valuable opportunity to assess the potential of river otters as biomonitors. The specific objectives of this study were to (1) document baseline contaminant levels in otters in a river system spanning an urbanization gradient; (2) test the utility and geographic scale of river otters as top trophic-level biomonitors of contaminant exposure for future assessments of broader ecological impacts of remediation and restoration efforts; and (3) evaluate the potential for health impacts from contaminants on river otters using published adverse effects thresholds.

## Methods

### Study area and scat sampling

The Green-Duwamish River in King County, Washington State, USA, is a 150-km river system originating in the Cascade Mountains about 48 km northeast of Mount Rainier and flowing into Puget Sound at the southern end of Elliott Bay in Seattle. The watershed drains approximately 1460 km^2^ into Puget Sound (King County & Washington State Conservation Commission, 2000).

**Fig. 1:**
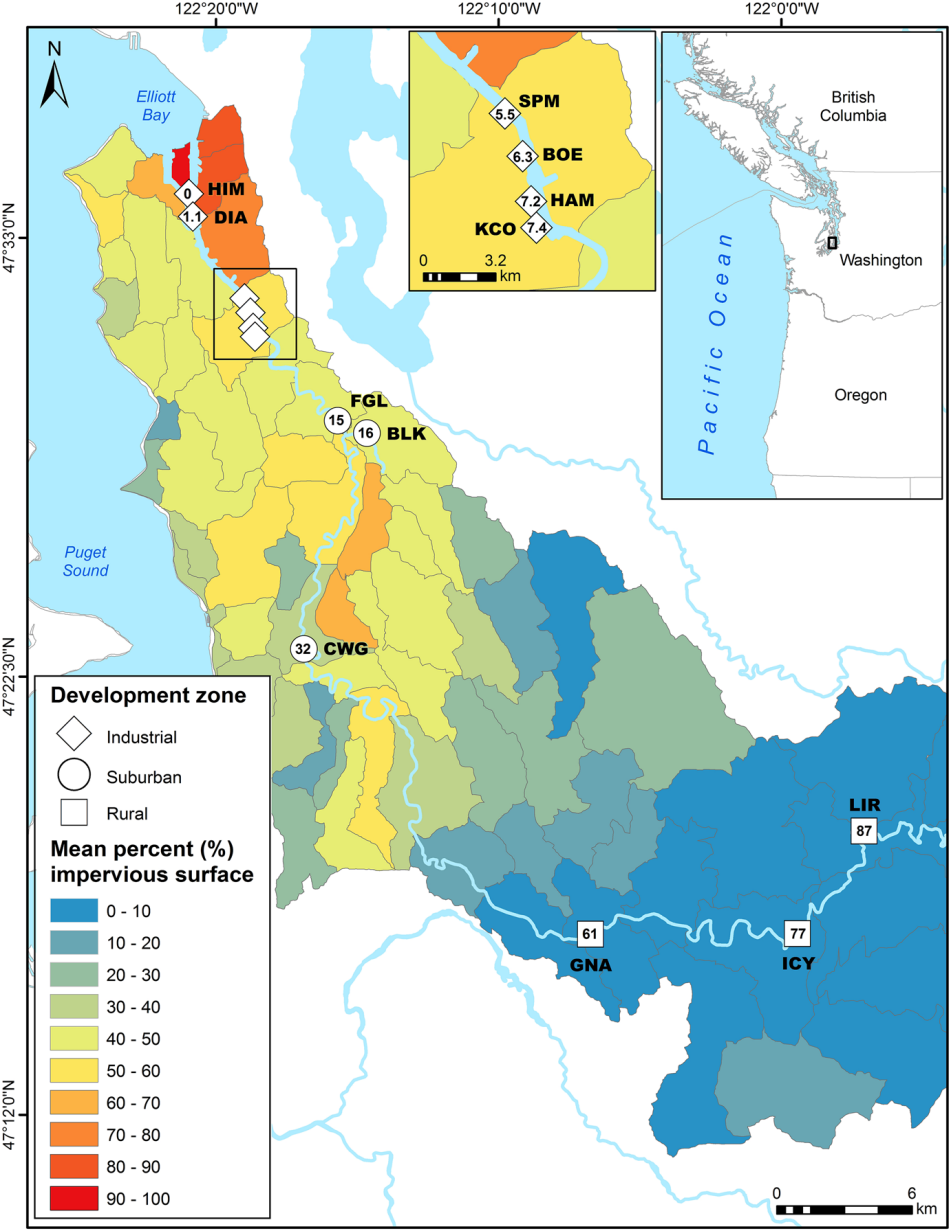
Locations and abbreviated names of river otter latrines where scats were collected (river kilometer inside development zone symbols) along the Green-Duwamish River in King County, Washington State, USA, for contaminant, stable isotope, and genetic analyses

All components of the study were conducted using river otter scat collected from 12 latrines along the Green-Duwamish River, ranging from river kilometer (rkm) 0 to 87 (Table 1, Fig. 1). The latrines were grouped spatially into three “development zones” based on impervious surface percentages in their adjacent watersheds: (1) industrial, with ≥ 50% impervious surface, which coincides with the LDW USEPA Superfund site (six latrines from rkm 0–7); (2) suburban, with 30–50% impervious surface (three latrines from rkm 14–32); and (3) rural, with 0–10% impervious surface (three latrines from rkm 61–87). Percent impervious surface in adjacent watersheds was determined by overlaying percent impervious surface land cover data from the 2016 National Land Cover Dataset (NLCD; MRLC, 2020) onto predefined watershed catchment areas adjacent to the Puget Sound shoreline. The NLCD Percent Developed Imperviousness dataset uses Landsat satellite data with a spatial resolution of 30 m (Homer et al., 2020). The watershed catchment areas were originally developed for another purpose (Stanley et al., 2016), and were determined to be of a size appropriate for use in this study (median area of 8.8 km^2^ or 3.4 mile^2^). Using these GIS layers, we calculated the average value (i.e., percent intensity) of impervious surface within each watershed adjacent to scat collection sites (latrines).

**Table 1:** Geometric mean concentrations and ranges of Σ_40_PCB, Σ_11_PBDE, Σ_6_DDT, and Σ_37_PAH, and arithmetic means of percent lipids (Lipids) and δ^15^N in river otter (*Lontra canadensis*) scat collected from 12 latrines in three development zones along the Green-Duwamish River in Washington State, USA; mean concentrations are reported as ng/g wet weight (ww) and mg/kg lipid weight (lw)

Development zone	Latrine	River KM	N	Lipids (%)	δ^15^N	Σ_40_PCB		Σ_11_PBDE		Σ_6_DDT		Σ_37_PAH	
						ww	lw	ww	lw	ww	lw	ww	lw
Industrial	HIM	0	3	1.54	12.6	130	9.1	2.6	0.18	2.1	0.14	**130**	9.1
						(91–270)	(4.7–24)	(1.3–9.0)	(0.071–0.80)	(1.2–5.1)	(0.071–0.45)	(110–160)	(5.2–12)
	DIA	1.1	2	0.601	13.6	**340**	**60**	**5.5**	**0.96**	3.9	**0.67**	110	20
						(84–**1400)**	(20–**180**)	(1.8–**17**)	(0.42–**2.2**)	(1.5–10)	(0.35–1.3)	(22–600)	(2.8–140)
	SPM	5.5	12	1.16	10.6	110	15	3.6	0.47	1.5	0.20	100	14
						(17–310)	(5.5–46)	(0.60–15)	(0.27–0.97)	(0.15–**12)**	(0.057–0.83)	(8.0–**4400**)	(2.5–80)
	BOE	6.3	2	0.448	10.9	100	24	2.6	0.62	2.1	0.49	**130**	**31**
						(63–160)	(21–27)	(1.9–3.6)	(0.60–0.64)	(1.6–2.7)	(0.45–0.54)	(39–450)	(6.5–**150**)
	HAM	7.2	8	1.26	11.0	93	11	3.0	0.34	2.3	0.27	92	11
						(31–400)	(2.8–43)	(0.96–9.4)	(0.086–1.0)	(0.91–4.5)	(0.076–1.7)	(13–2500)	(0.61–120)
	KCO	7.4	8	1.24	10.3	130	12	5.9	0.58	**5.4**	0.54	62	6.1
						(62–340)	(3.1–48)	(2.6–17)	(0.074–1.2)	(2.5–11)	(0.099–1.5)	(16–1100)	(0.45–56)
	All industrial		35	1.16	11.0	**120**	**14**	**3.8**	**0.45**	**2.5**	**0.29**	**93**	**11**
						(17–**1400**)	(2.8–**180**)	(0.6–**17**)	(0.071–**2.2**)	(0.15–**12**)	(0.057–1.7)	(8.0–**4400**)	(0.45–**150**)
Suburban	FGL	14.5	7	0.507	6.54	12	2.7	1.6	0.35	0.57	0.13	18	3.9
						(5.9–31)	(1.2–4.4)	(0.27–7.2)	(0.10–1.0)	(0.082–4.3)	0.029–0.84)	(5.1–79)	(0.95–11)
	BLK	16.1	7	0.710	8.70	23	4.0	2.5	0.44	0.84	0.15	39	6.8
						(10–33)	(1.9–9.4)	(0.73–4.5)	(0.18–0.87)	(0.22–4.6)	(0.072–0.31)	(25–120)	(2.1–21)
	CWG	32.2	7	0.575	6.91	10	2.2	1.2	0.26	1.2	0.26	11*	2.3*
						(2.0–71)	(0.91–11)	(0.17–8.4)	(0.078–1.3)	(0.16–11)	(0.073–**1.9**)	(1.2–43)	(0.41–7.5)
	All suburban		21	0.597	7.38	14	2.9	1.7	0.34	0.84	0.17	19*	3.9*
						(2.0–71)	(0.91–11)	(0.17–8.4)	(0.078–1.3)	(0.082–11)	(0.029–**1.9**)	(1.2–120)	(0.41–21)
Rural	GNA	61.2	4	0.304	3.13	1.2	0.44	0.14*	0.050*	0.096*	0.034*	3.4	1.2
						(0.71–1.6)	(0.25–1.3)	(0.098–0.19)	(0.031–0.078)	(0.080–0.016)	(0.019–0.063)	(2.0–9.6)	(0.54–2.3)
	ICY	77.2	3	0.284	1.85	1.2	0.50	0.12*	0.047*	0.12*	0.048*	2.6	1.1
						(0.84–2.0)	(0.17–0.99)	(0.070–0.21)	(0.022–0.10)	(0.070–0.16)	(0.032–0.074)	(1.0–13)	(0.20–6.4)
	LIR	86.9	6	0.852	4.92	3.4	0.67	0.36*	0.073*	0.30*	0.060*	5.5	1.1
						(1.0–20)	(0.42–1.4)	(0.13–1.8)	(0.041–0.18)	(0.070–5.1)	(0.029–0.16)	(2.7–28)	(0.88–1.4)
	All rural		13	0.552	3.66	1.9	0.55	0.21*	0.059*	0.17*	0.048*	4.0	1.1
						(0.71–20)	(0.17–1.4)	(0.070–1.8)	(0.022–0.18*)*	(0.070–5.1)	(0.019–0.160)	(1.0–28.0)	(0.20–6.4)

River KM begins with 0 at the mouth of the Green-Duwamish River and increases moving upstream (see also Fig. 1). Development zones are based on impervious surface land cover as defined in the methods. Maximum latrine and development zone contaminant concentrations are indicated in bold. Five naphthalene analytes (NPHs = NPH, C_1_NPH, C_2_NPH, C_3_NPH, C_4_NPH) were excluded from PAH analyses because laboratory blanks contained NPHs at high enough concentrations to account for observed levels in scat samples

^*^Mean and associated range include at least one less than the lower limit of quantitation (< LOQ) replacement value randomly generated from the range of < LOQs for the relevant contaminant category

Preliminary surveys were conducted in winter and spring 2016 by foot along accessible areas of the Green-Duwamish River to identify latrines, conspicuous terrestrial sites where river otters repeatedly defecate and leave “anal jellies” with high site fidelity (Gorman et al., 2006). Groupings of latrines were then visited daily for 13 to 22 days to identify and collect fresh scat. On the first day of a latrine visit, all existing scat was sprinkled with glitter. On each of the subsequent daily latrine visits, scat lacking glitter could be characterized as less than 24 h old and was sampled as described below; any uncollected fresh scat was also marked with glitter. In 2016, scat was collected in all three development zones during July–August and September–October, terminating with the onset of fall rains, which can contaminate scat samples either directly or via runoff from surrounding substrate. Scat was sampled at all times of day based on the order of visitation to the latrines. In 2017, scat was collected in the industrial and suburban zones during May and September. In an effort to improve DNA extraction yield above 2016 levels, a team was deployed to simultaneously sample scat at all targeted latrines in the early morning hours prior to any exposure of each site to direct sunlight.

Scat processing began in the field with DNA sampling, following the protocol of Klütsch and Thomas (2018). Briefly, fresh scat was swabbed by gently passing a sterile cotton tip applicator over the mucus layer of the scat, sampling approximately 3–6 cm^2^ of the scat surface, as scat size allowed. The swab tip was rinsed in a 1.5 ml microtube of DNA lysis buffer solution (4 M urea, 0.2 M NaCl, 0.5% n-lauroyl sarcosine, 10 mM 1,2-ethylenediaminetetraacetic acid (EDTA), 0.1 M Tris–HCl, pH 8.0), then the swab was passed over the scat surface a second time. The tip of the applicator was snapped off and sealed into the same 1.5-ml microtube.

After swabbing, fresh scat were inspected visually and categorized as containing either fish hard parts, crustacean hard parts, or an obvious mixture of the two. Scat were then collected whole with wooden craft sticks or twigs and placed in chemically rinsed 60 ml clear or amber glass jars. DNA sample microtubes and scat jars were stored within 30 min in a cooler with ice packs. DNA samples were transferred daily to a 4 °C refrigerator until they were shipped to the National Wildlife Research Center, Environment and Climate Change Canada, Carleton University (Ottawa, ON, Canada) for subsequent analyses. Whole scat samples for contaminant analysis were transferred daily to a − 20 °C freezer until they were delivered on ice to the NOAA Northwest Fisheries Science Center, Seattle, WA and stored at − 80 °C prior to analyses.

### Contaminant analysis

Scat samples (*n* = 69) analyzed for contaminants included 35 (51%) from the six latrines in the industrial zone, 21 (30%) from the three latrines in the suburban zone, and 13 (19%) from the three latrines in the rural zone (Table 1, Fig. 1). Approximately 2–2.5 g from each scat were extracted and analyzed for POPs and PAHs, including 40 PCBs, 11 PBDEs, six DDTs, eight CHLDs, hexachlorobenzene (HCB), and 37 PAHs (17 low molecular weight, LMW, and 20 high molecular weight, HMW) using an established gas chromatography/mass spectrometry (GC/MS) method (Sloan et al., 2014). Specifications for instrumentation parameters, standards, supplies, and materials used in POP GC/MS runs are detailed in Sloan et al. (2014). Briefly, this method is comprised of three steps: (1) a dichloromethane extraction using high pressure and high temperature in an accelerated solvent extractor, (2) a two-step cleanup on a gravity flow column containing silica/aluminum to remove polar compounds followed by size-exclusion high-performance liquid chromatography (HPLC) to remove lipids and other biogenic compounds, and (3) separation and quantitation of POPs using GC/MS with selected-ion-monitoring (SIM). For the PAH analyses, the same extracts that were analyzed for POPs were analyzed on the GC/MS system in a separate run (see Sloan et al., 2014 for details of the PAH GC/MS method). A subsample of each pre-cleaned extract was used to determine percent lipids gravimetrically (Sloan et al., 2014).

A solvent (dichloromethane) blank and two National Institute of Standards and Technology standard reference materials (SRM 1974c mussel and SRM 1947 fish tissue) were analyzed with each sample batch as part of the laboratory’s performance-based quality assurance (QA) program (Sloan et al., 2019). All QA samples met established laboratory criteria. For example, the concentrations of ≥ 70% of individual analytes that were measured in the NIST SRMs were within 30% of either end of the 95% confidence interval range of the published NIST certified POP or PAH concentration of that analyte. Method blanks contained no more than five analytes that exceeded two times the lower limit of quantitation (LOQ). The percent recoveries of the surrogate standards for all field and quality assurance samples ranged from 68 to 128% and were within the laboratory criteria range of 60–130%. The LOQ values for individual PCBs, PBDEs, DDTs, CHLDs, and HCB were based on sample mass and instrument performance for each batch of samples (Sloan et al., 2014) and ranged from < 0.055 to < 0.51 ng/g, wet weight. LOQ values for PAHs ranged from < 0.16 to < 1.3 ng/g, wet weight.

POPs and PAH data are presented as summed values for each contaminant class, expressed as nanogram per gram of scat wet weight (ng/g ww) and on a lipid basis, as milligram of contaminant per kilogram of scat lipid weight (mg/kg lw), to facilitate comparisons with other studies. Summed PCBs were calculated by summing detected concentrations of congeners 17, 18, 28, 31, 33, 44, 49, 52, 66, 70, 74, 82, 87, 95, 99, 101/90, 105, 110, 118, 128, 138/163/164, 149, 151, 153/132, 156, 158, 170, 171, 177, 180, 183, 187/159/182, 191, 194, 195, 199, 205, 206, 208, and 209. All PCB congeners were detected in at least one sample, and at least three congeners were detected in each sample. Summed PBDEs were calculated by summing detected concentrations of the congeners 28, 47, 49, 66, 99, 100, 153, 154, and 155. PBDE congeners 85 and 183, although measured, were not detected in any of the samples analyzed. The remaining PBDE congeners were detected in at least one sample. For the five samples (7%) in which no PBDE congener was measured above the LOQ, a value within the range of LOQs (0.056 and 0.17 ng/g) was randomly assigned as the Σ_11_PBDE concentration for statistical analysis. Summed DDTs were calculated by summing detected concentrations of the congeners *o,p′*-DDD, *o,p′*-DDE, *o,p′*-DDT, *p,p′*-DDD, *p,p′*-DDE, and *p,p′*-DDT. All DDT congeners were detected in at least one sample. For the eight samples (12%) in which no DDT congener was measured above the LOQ, a value within the range of LOQs (0.055 and 0.17 ng/g) was randomly assigned as the Σ_6_DDT concentration for statistical analysis. Summed CHLDs were calculated by summing detected concentrations of the congeners *cis*-chlordane, *trans*-chlordane, heptachlor, heptachlor epoxide, *cis*-nonachlor, *trans*-nonachlor, nonachlor III, and oxychlordane. Heptachlor was not detected in any of the samples analyzed. All other CHLDs were detected in at least one sample. For the eight samples (12%) in which no CHLD congener was measured above the LOQ, a value within the range of LOQs (0.056 and 0.50 ng/g) was randomly assigned as the Σ_8_CHLD concentration. HCB, also measured, was not detected in seven samples (10%). For the seven samples (10%) in which no HCB was detected above the LOQ, a value within the range of LOQs (0.056 and 0.15 ng/g) was randomly assigned. Summed PAHs were calculated from 37 PAH analytes (supplementary information, Table S1). Naphthalene and C1- through C4-naphthalenes were excluded from analyses; they were commonly detected in scat samples, but also occurred in solvent blanks, presumably related to uncontrollable ambient sources during processing. All PAH analytes were detected in at least one sample. For the single sample (1%) in which no PAH analytes were detected, a value within the range of LOQs (0.16 and 1.3 ng/g) was randomly assigned as the Σ_37_PAH concentration for statistical analysis. Concentrations were uniformly low or percent non-detects (ND) high for *alpha*-, *beta*-, and *gamma*-hexachlorocyclohexane (HCHs; 94% ND), dieldrin (42% ND), and mirex (94% ND); values from samples with detections for these compounds were used in the discussion only for comparative purposes. Observed contaminant values are presented as geometric means to minimize the disproportionate effects of extreme values (arithmetic means are presented in supplementary information; Table S2, Table S3).

### Nitrogen stable isotope analysis

Scat samples were also analyzed for the stable isotope ratio of nitrogen ^15^N:^14^N to provide quantitative insight into the role of diet and prey trophic levels on river otter contaminant levels (Caut et al., 2009). Frozen scat samples were freeze-dried and ground to a fine powder using a micro ball-mill. Each sample was weighed into a tin capsule and was combusted in a Thermo Fisher Scientific Flash 2000 Elemental Analyzer coupled with the Conflo IV interface and analyzed using a Delta V Advantage Isotope Ratio Mass Spectrometer (Gates et al., 2020). Values were calibrated against internal laboratory standards (aspartic acid and ^15^N-enriched histidine), which were analyzed after every 10 field samples.

Quality assurance measures for stable isotope ratios included the analysis of both continuing calibration standards and a fish tissue, SRM 1946 (National Institute of Standards and Technology, Gaithersburg, MD, USA), with each batch of samples (Sloan et al., 2019). Continuing calibration standards were run every 10 field samples, whereas SRM 1946 was run between every 20 samples. Isotope values for continuing calibration standards and SRM 1946 were within 0.30‰ of the values calibrated against international standards for δ^15^N.

Nitrogen stable isotopes were expressed in standard delta notation (δ^15^N), as

$$\delta \left(\permil \right)={10}^{3} \left[\left({R}_{sample}{/R}_{standard}\right)-1\right],$$

where *R* is the ratio of ^15^N:^14^N isotopes. We expressed stable isotope ratios in units of permil (‰—parts per thousand) and as relative to the international standard of atmospheric nitrogen for δ^15^N.

### Genetic analysis

A total of 152 scat samples underwent genetic analysis to assess the number of individual river otters represented by our scat samples and the spatial extent of movement of otters between latrines (for detailed methods, see supplementary information). DNA quality was assessed by amplifying the mitochondrial DNA (mtDNA) control region. Samples with successful mtDNA screening were PCR-amplified at 14 microsatellites designed for North American river otters (Beheler et al., 2004, 2005; Mowry et al., 2011) in single- and multiplexes. Quality assurance measures included the statistical analyses of genotyping errors, excess homozygosity, null alleles, and allelic dropout rates. Metrics of genetic diversity and pairwise relatedness are detailed in the supplementary information.

### Data analysis

We applied multiple linear regression (R Core Team, 2020) to identify the potential effects of three main factors on contaminant concentrations for three major POP classes (PCBs, PBDEs, and DDTs) and PAHs. The three predictor variables were as follows: (1) location, either as development zone (industrial, suburban, rural) or individual latrine site; (2) δ^15^N; and (3) lipid content (percent lipid). The two levels of categorical location data—development zone or latrine site—were investigated (exclusive of each other in models) to assess at what scale latrine location impacted contaminant concentrations in otter scat. δ^15^N was included as a continuous proxy variable for diet because it was a strong predictor of visually-determined diet category (fish, crustacean or mixed; see Fig. S1 in supplementary information). Lipid content was included as it can affect the concentrations of contaminants accumulated (West et al., 2017). All contaminant data were natural-log (ln) transformed to meet assumptions of normality and homogeneity of group variances, and percent lipid data were ln-transformed to ensure a linear relationship with contaminant concentrations. Additive and interactive effects were evaluated; however, due to limited degrees of freedom, only models with up to three factors were considered. Akaike Information Criterion corrected for small sample size (AICc) was used to identify the best model to parsimoniously explain the variation in concentrations of contaminants (Akaike, 1974; Burnham & Anderson, 2002). We selected the model with the lowest AICc score as the most parsimonious model, unless a simpler model existed with a ΔAICc score less than or equal to 2 compared to the more complex model (Burnham & Anderson, 2002). For the best fit model for each contaminant class, multiple comparisons between estimated means were conducted using the Sidak adjustment (emmeans package; Lenth, 2020). Test results for pairwise comparisons were considered statistically significant at *p* ≤ 0.05. Because CHLD and HCB concentrations were significantly correlated with levels of DDTs (CHLD: r^2^ = 0.7464, *p* < 0.0001; HCB: *r*^2^ = 0.6002, *p* < 0.0001), we opted not to run additional models but rather inferred that similar variables are involved in driving CHLD and HCB concentrations as those that drive DDT concentrations.

Although samples were collected over two calendar years and across multiple seasons, these factors were not considered in our analysis due to limited sample sizes. One scat sample was identified as an outlier for lipid content using Grubb’s test and excluded from analysis (12.4% compared to a range of 0.13–5.5% and median of 0.6% for all other samples). Field notes revealed that this sample contained globular, fatty substances and fish roe, atypical of scat samples in this study.

### Toxicological significance

To assess toxicological significance of the concentrations of Σ_40_PCBs measured in river otter scat in our study, we compared our data to the following threshold values established in Mason et al. (1992) and Mason and Macdonald (1993a), and also referenced in numerous other river otter contaminant studies (e.g., Elliott et al., 2008; Guertin et al., 2010; Huang et al., 2018; Lemarchand et al., 2007; Mason & Macdonald, 1993b; Ruiz-Olmo et al., 2000): a *critical level* of ΣPCBs > 16 mg/kg lw; a *level of concern* of ΣPCBs 9–16 mg/kg lw; *maximum allowable level* of 4–9 mg/kg lw; and *no effect level* of < 4 mg/kg lw. These thresholds were developed using a two-stage model that uses empirical data on the relationships between contaminant levels in otter feces and prey, then prey and otter liver tissue (Mason et al., 1992). Scat PBDE concentrations were converted to liver tissue values (mg/kg lw) following La Guardia et al. (2020) and compared to threshold values discussed therein. No threshold values are currently published for river otters for any of the remaining POP classes or PAHs.

## Results

### Spatial variation in contaminant concentrations

Contaminant concentrations varied widely across contaminant class and location. Overall, the rank order of contaminant classes by concentration was PCBs ≈ PAHs >  > PBDEs > DDTs ≈ CHLDs > HCB (see Table 1 for geometric means of PCBs, PAHs, PBDEs, and DDTs; see Table S2 for arithmetic means of these contaminants; see Table S3 for geometric and arithmetic means of CHLDs and HCB). All 69 scat samples had detectable levels of Σ_40_PCBs, while most samples had detectable levels of Σ_37_PAHs (68 scats), Σ_11_PBDEs (66 scats), Σ_6_DDTs (63 scats), Σ_8_CHLDs (63 scats), and HCB (64 scats). Undetected concentrations were primarily from scats collected in the rural zone, except for PAHs from a suburban zone scat sample and HCB from one suburban zone and two industrial zone scat samples. In general, concentrations of all contaminant classes increased with increasing urbanization, represented by development zone. Geometric mean Σ_40_PCB concentrations were the most variable of the contaminant classes, ranging 26-fold across development zones, from 0.55 mg/kg lw in the rural region to 14 mg/kg lw in the industrial region. Similar though less-pronounced patterns were found for all other contaminant classes. Geometric mean (mg/kg lw) Σ_37_PAH and Σ_11_PBDE concentrations ranged 10- and 8.3-fold across development zones, respectively, while Σ_6_DDT, Σ_8_CHLD, and HCB concentrations varied less, ranging 6.6-, 2.9-, and 1.5-fold, respectively.

Contaminant sums were dominated by a limited number of congeners for all contaminant classes. PCB153 and PCB138 accounted for approximately 25% and 20%, respectively, of Σ_40_PCBs across all development zones. PBDE47 ranged from 75 to 92% of Σ_11_PBDEs across development zones, while *p,p′*-DDE comprised 73–99% of Σ_6_DDTs across development zones. PAH analyte profiles were more variable across development zones: in the industrial and suburban zones, PHN and FLA/PYRs each account for approximately 25–30% of Σ_37_PAHs, whereas in the rural zone, PHN comprises 88% of all PAHs (see Table S1 for PAH abbreviations).

**Fig. 2:**
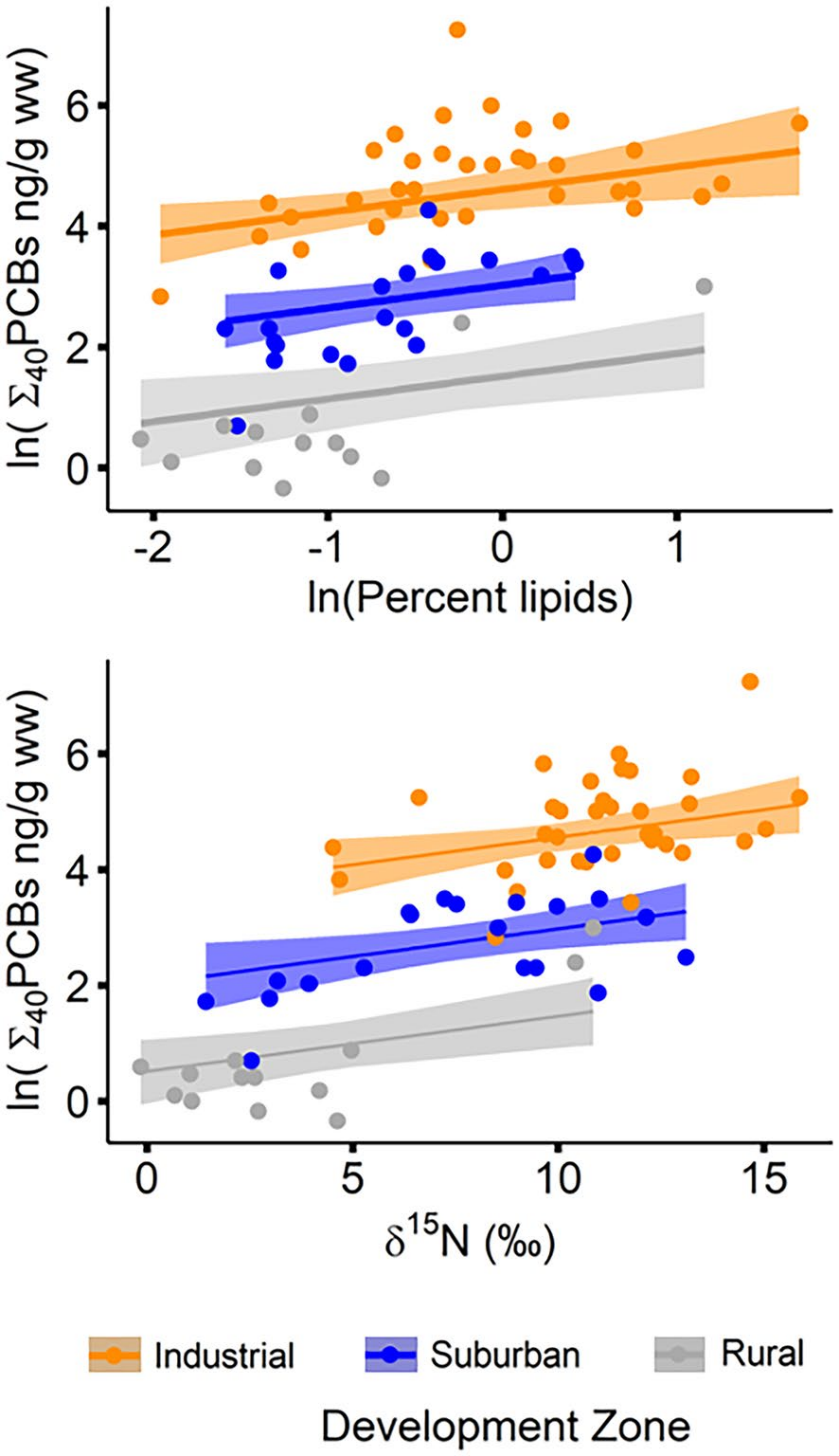
Relationship between Σ_40_PCB concentrations (ng/g ww) and lipid content and δ^15^N in river otter scat from the industrial (orange), suburban (blue), and rural (gray) zones of the Green-Duwamish River, Washington, USA; shaded regions represent ± 95% CI, actual data plotted in solid symbols

For all four contaminant classes modeled (PCBs, PBDEs, DDTs, and PAHs), pollutant concentrations increased with increasing development along the rural-suburban-industrial gradient and with increasing lipid content, but the importance of these and other factors varied by contaminant class. Concentrations of Σ_40_PCBs in river otter scat were best predicted by the model that included development zone, lipid content, and δ^15^N (supplementary information, Table S4; adjusted *r*^2^ = 0.852). Comparing only models with single factors, the model with development zone alone accounted for considerably more variation (adjusted *r*^2^ = 0.774) than single-factor models with δ^15^N alone or lipid content alone (adjusted *r*^2^ = 0.630 and 0.360, respectively), indicating that development zone was the most important factor for predicting Σ_40_PCBs. The next two best supported models included development zone and either δ^15^N or lipid content, and both models had nearly equal support (Table S4; adjusted r^2^ = 0.840 and 0.840, ΔAICc = 0.16). Estimated mean Σ_40_PCBs were significantly different between all development zones with levels increasing along the rural-suburban-industrial gradient (Table 2, Fig. 2; *p* values < 0.0001 for all comparisons).

**Table 2:** Predicted mean concentrations, standard errors (SE), and 95% confidence intervals (CI) for the best-fitting model for each POP class and PAHs (ln values)

POP class	Covariates	Development zone	Estimated mean	SE	95% CI	
ln∑_40_PCBs	δ^15^N = 8.52 ln(lipids) = − 0.497	Industrial	4.42	0.139	4.08–4.76	c
		Suburban	2.84	0.156	2.45–3.22	b
		Rural	1.33	0.243	0.734–1.92	a
ln∑_11_PBDEs	ln(lipids) = − 0.497	Industrial	1.095	0.134	0.770–1.42	b
		Suburban	0.676	0.168	0.265–1.09	b
		Rural	− 1.271	0.221	− 1.81 to − 0.729	a
ln∑_6_DDTs	ln(lipids) = − 0.497	Industrial	0.716	0.168	0.381–1.05	b
		Suburban	0.141	0.212	− 0.281–0.564	b
		Rural	− 1.20	0.309	− 1.81 to − 0.579	a
ln∑_37_PAHs	δ^15^N = 8.52	Industrial	4.04	0.233	3.57–4.50	b
		Suburban	3.20	0.264	2.67–3.72	ab
		Rural	2.36	0.403	1.56–3.17	a

Estimates for contaminant concentrations are shown by development zone based on the grand mean of percent lipid content (0.608; ln-transformed =  − 0.497) and/or grand mean of δ^15^N = 8.52. Pairwise comparisons were conducted on estimated marginal means using the Sidak adjustment for multiple comparisons. For each POP class and PAHs, groups with the same lowercase letter were not significantly different from each other. For additional comparisons using mean lipid content in each of three development zones, see Table S5

**Fig. 3:**
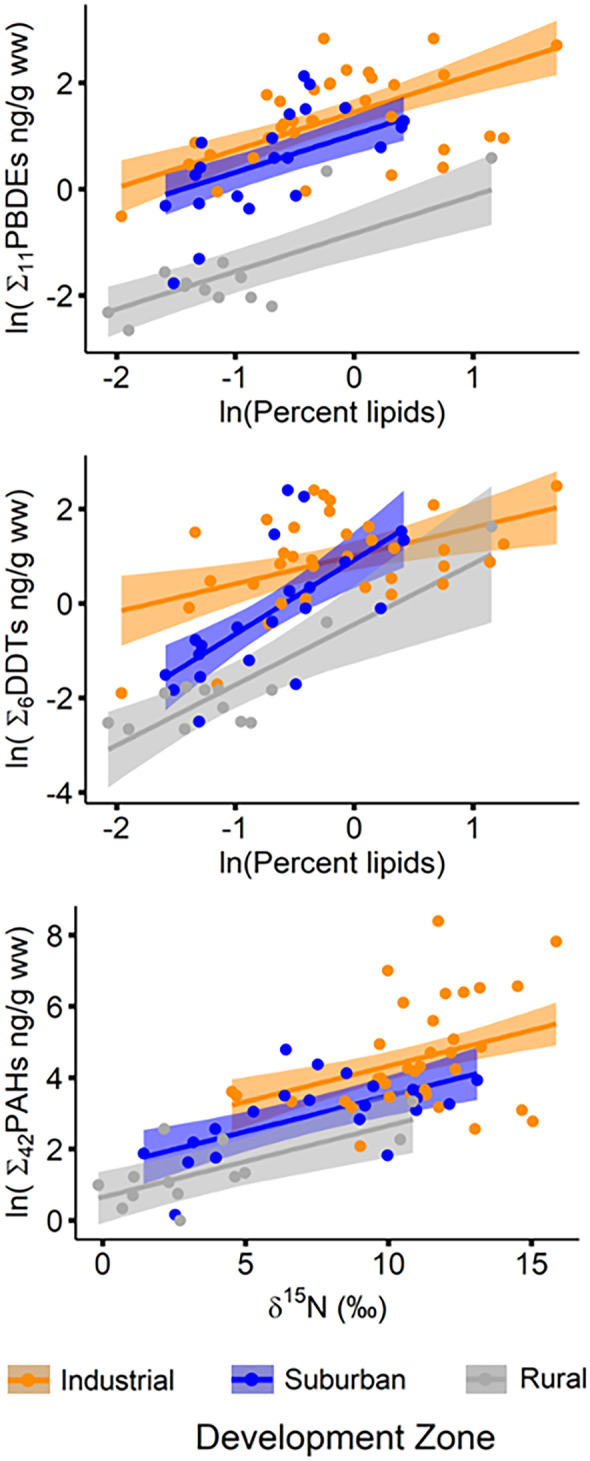
Relationship between lipid content or δ^15^N and estimated Σ_11_PBDE, Σ_6_DDT, and Σ_37_PAH concentrations (ng/g ww) in river otter scat from the industrial (orange), suburban (blue), and rural (gray) zones of the Green-Duwamish River, Washington, USA; shaded regions represent ± 95% CI, actual data plotted in solid symbols

Concentrations of Σ_11_PBDEs were best predicted by development zone and lipid content, which together accounted for 72% of the variation (Table S4). Although two other models had lower AICc scores, this was the most parsimonious model to explain the data (Table S4; adjusted *r*^2^ = 0.722, ΔAICc = 0.9). In single-factor models with development zone or lipid content, development accounted for a greater percent of the variation than lipid content (*r*^2^ = 0.587 vs. 0.396), again highlighting the relative importance of development zone as a factor influencing contaminant concentrations in otter scat. Estimated mean Σ_11_PBDE concentrations were significantly lower in otter scat collected from the rural zone compared to the suburban and industrial zones, which were not significantly different from each other (Table 2, Fig. 3a).

The Σ_6_DDT concentrations were best explained by development zone, lipid content, and a development zone × lipid content interaction term (Table S4; adjusted *r*^2^ = 0.653). Similar to PBDEs, estimated mean Σ_6_DDT levels in the rural zone were significantly lower than the suburban and industrial zones, which were not significantly different from each other (Table 2, Fig. 3b). However, these results are based on a grand mean lipid content value (ln-transformed =  − 0.497). Due to the interaction between development zone and lipid content, differences in Σ_6_DDT concentrations across development zones depend on the lipid content of the samples being compared (supplementary information, Table S5). At lower lipid content, consistent with the mean lipid content of otter scat from the rural (ln-transformed mean =  − 1.0) and suburban (ln-transformed mean =  − 0.7) zones, significant differences in mean Σ_6_DDT concentrations were observed between all development zones; at higher lipid content, consistent with the industrial zone mean (ln-transformed mean =  − 0.17), mean Σ_6_DDT levels were significantly lower in otter scat from the rural zone, but no differences existed between the suburban and industrial zones (similar to the grand mean pattern). Another highly supported model included δ^15^N as a third factor in addition to development zone and lipid content (Table S4; ΔAICc = 1.3), again highlighting the value of δ^15^N in predicting contaminant concentrations.

Levels of Σ_37_PAHs showed similar patterns to the POPs evaluated; however, the predictive power of PAH models was notably weaker. Development zone and δ^15^N best explained Σ_37_PAH levels (Table S4; adjusted r^2^ = 0.561), followed closely by development zone and lipid content (adjusted *r*^2^ = 0.559; ΔAICc = 0.2). Though the model with the lowest AICc included development zone and both lipid content and δ^15^N, this three-factor model was less parsimonious (Table S4). Estimated mean Σ_37_PAH levels were significantly higher in the industrial zone compared to the rural zone, while the suburban zone was not significantly different from either of the other two zones (Table 2, Fig. 3c).

### Diet factors

Lipid content varied in samples from 0.126 to 5.50% and was significantly higher in scat collected in the industrial zone than in those samples obtained in suburban and rural zones (one-way ANOVA, *F* = 7.3571, *p* < 0.01), which were not different from each other (see supplementary information, Table S6). δ^15^N varied in samples from − 0.149 to 15.9‰ and significantly increased across all three zones along the rural-suburban-industrial gradient (one-way ANOVA, *F* = 29.247, *p* < 0.001; Table S6). The patterns of δ^15^N in river otter scat also were representative of the categorical field characterizations of prey hard parts visible during scat collection and aligned with the general trophic levels associated with these prey items, with significant differences across all diet categories (*p* < 0.05 for all comparisons; Fig. S1). In the industrial zone, 91% of scats were composed primarily of fish; in the suburban zone, scats were split nearly in thirds among the three diet categories (fish, crustaceans, mixed); and in the rural zone, 70% of scats were composed of crustaceans.

### Toxicological significance

**Fig. 4:**
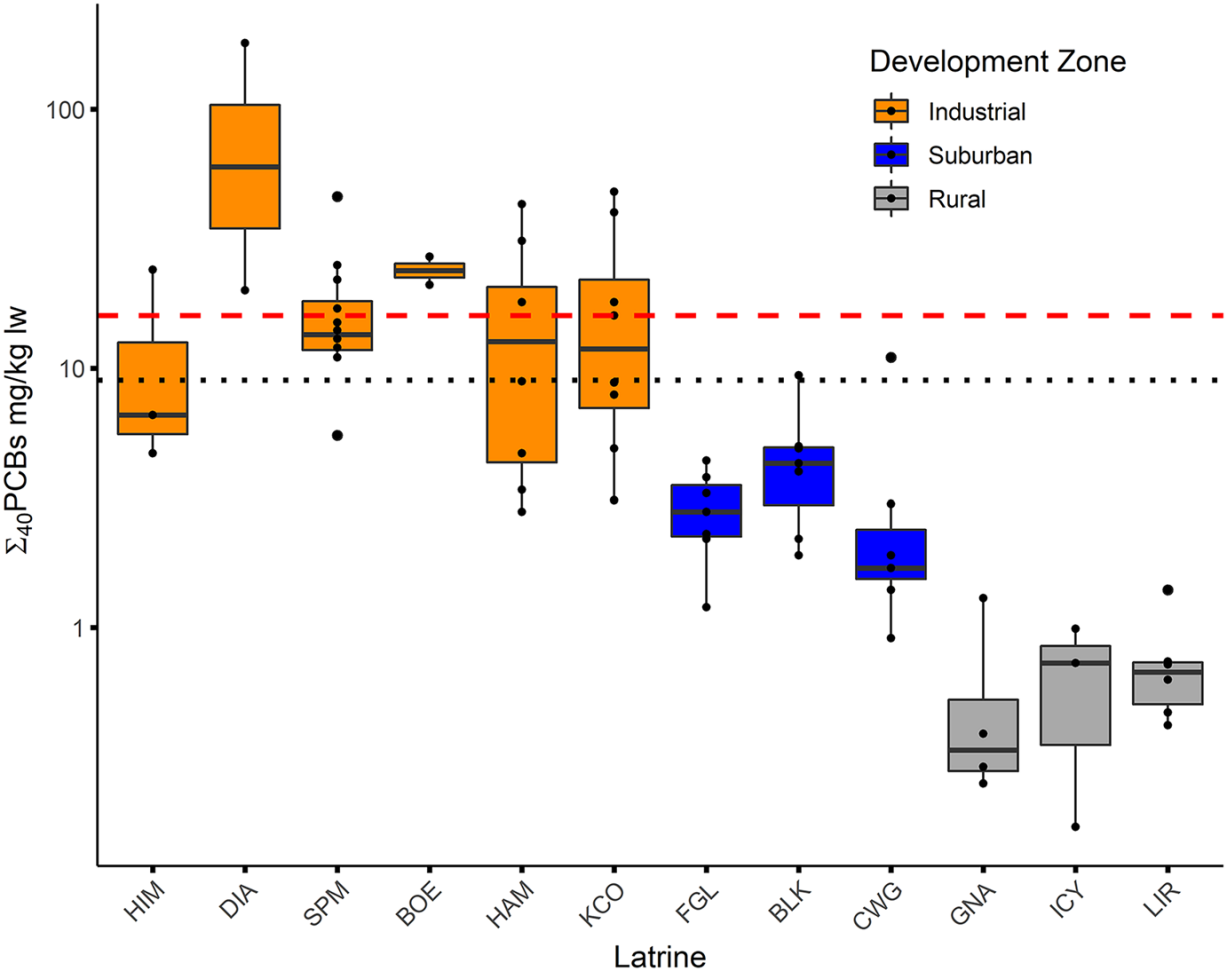
Median concentrations of ∑_40_PCBs (mg/kg lw) in river otter scat collected at each of 12 latrines located in three different development zones along the Green-Duwamish River, Washington, USA, as compared to published values for level of concern (9 mg/kg lw; black dotted line) and critical level (16 mg/kg lw; red dashed line)

In the industrial zone, 46% of otter scat samples analyzed in the current study had Σ_40_PCB levels above the critical threshold of 16 mg/kg lw and another 23% fell within the level of concern (9–16 mg/kg lw; Fig. 4). None of the scat samples from the suburban zone exceeded the critical PCB threshold value (16 mg/kg lw), though 10% reached the level of concern (9–16 mg/kg lw). All samples in the rural zone fell into the no effect level (< 4 mg/kg lw). The highest concentration of Σ_40_PCBs for an individual scat was 180 mg/kg lw at DIA, one of the latrines nearest the mouth of the river in the industrial zone (Table 1, Fig. 1).

The ∑_11_PBDE liver concentration estimates were predominantly far below the no-observed-adverse-effect-level (NOAEL) of 1.19 mg/kg lw (liver tissue) for juvenile mink established by Zhang et al. (2009). The ∑_11_PBDE liver concentration estimate in one industrial zone scat sample reached the NOAEL, and one additional scat in the industrial zone exceeded half the NOAEL (supplementary information, Table S7).

### Genetics

Of 152 scat swabs, 27 (18%) successfully amplified at ≥ 14 microsatellite loci. This low success rate prevented estimation of the total number of unique otters sampled by the 152 swabs. In addition, the small sample size precluded the analysis of genetic data to estimate local otter population size, assess the degree of transit between development zones, whether otter movement may influence patterns of contaminant concentrations in scat, and whether either of these factors may be influenced by sex. Despite these limitations, genetic results from this study provide descriptive insight and ancillary information relevant to our interpretation of contaminants analysis, as well as cautionary information regarding sampling conditions (see supplementary information).

Eighteen different individuals were identified among the 27 amplified samples. Twelve (67%) were male and six (33%) were female. Of the six individuals that were re-sampled (multiple scats attributed to the same individual), two were detected repeatedly at the same latrine (one male, one female), two were detected at different latrines within the industrial zone (one male, one female; maximum distance 5.5 km) and two were detected at latrines in different development zones (one male at 32 km between scats [CWG to HIM, Fig. 1]; one female at 25 km between scats [CWG to KCO, Fig. 1]). All recaptures occurred within years, and there were no temporal patterns related to distance. Scats from the male detected 32 km apart were collected 49 days apart, while scats from the female detected 25 km apart were collected 10 days apart. Meanwhile, scats from otters detected within the same development zone and even at the same latrine varied in collection windows from 0 days (same collection event, scats were far apart and/or different in appearance enough to be considered separate) to 112 days.

Results for excess homozygosity, null alleles, allelic dropouts, genetic diversity, and pairwise relatedness are presented in the supplementary information.

## Discussion

### Spatial variation in contaminant exposure

The Lower Duwamish Waterway (LDW, industrial zone) of the Green-Duwamish River is a US Superfund site, heavily contaminated with PCBs, PAHs and other pollutants from over a century of use as the primary industrial corridor for Seattle, WA (USEPA, 2014). Since Superfund designation, elevated levels of PBDEs have also been detected in invertebrates and fish in the LDW, possibly associated with discharges of wastewater effluent (Lanksbury et al., 2013; O’Neill et al., 2015; West et al., 2017). At the other extreme, the upstream rural zone of the Green-Duwamish River is characterized by light agricultural use and a nearly continuous series of state and county parks established below a highly protected catchment area that serves as a source of drinking water for the nearby city of Tacoma, WA.

As expected, mean concentrations of all contaminant categories in otter scat declined significantly and sometimes dramatically across this urbanization gradient (Table 2, Figs. 2 and 3). The Σ_40_PCB levels were significantly different among all three development zones, while Σ_11_PBDEs were significantly lower only in the rural zone. This may reflect the nature of pathways bringing PBDEs into the Puget Sound basin, with direct air deposition from anthropogenic sources and residential wastewater broadening inputs across both the industrial and suburban zones (King County, 2013; Osterberg & Pelletier, 2015). Concentrations of Σ_37_PAHs were significantly higher in the industrial zone compared to the rural zone, likely reflecting the concentration of emissions and creosote structures in industrial and urban areas that have historically been major sources of PAHs in the region (King County, 2013; Osterberg & Pelletier, 2015). The Σ_6_DDT concentrations were low overall and, unlike for other contaminants, there was an interaction between Σ_6_DDT and scat lipid content. Scat in the industrial zone had relatively high Σ_6_DDT levels regardless of lipid content, whereas Σ_6_DDT levels in the suburban and rural zones approached those of the industrial zone only as lipid levels increased. It is unclear what may be driving this pattern. Given the relative degree of environmental contamination, DDT levels may be higher in prey across all lipid ranges in the industrial zone, while accumulating only in higher-lipid content prey in the suburban and rural zones.

Although our data show clear regional patterns in contaminant concentrations along the Green-Duwamish River gradient, differences were not observed among individual latrine sites, suggesting that otters are not useful for monitoring finer spatial scale gradations of contaminant exposure. The Lower Duwamish Waterway (industrial zone) and the Middle Green River (rural zone) represent extreme end-points of an urbanization gradient, so between-zone differences are likely to override within-zone variability. In addition, many factors can contribute to the dilution of the predictive power of individual latrine sites, including the mobility of otters and prey (discussed in more detail below in “River otters as biomonitors”). River otters are aquatic foragers yet use terrestrial latrine sites to defecate; even with less-mobile prey, this act alone may uncouple the spatial relationship between these events. River otters have displayed food retention times of several hours (Ormseth & Ben-David, 2000; White et al., 2007), providing ample time to move from the immediate environment of a foraging event and even the nearest latrines. In the LDW, sediment contamination “hot spots” have been documented on the scale of 3–5 acres (0.01 to 0.02 km^2^), and for pollutants entering any geographic zone of the river through storm- and wastewater outfalls, there are likely to be small-scale gradations in sediment contamination that cannot be captured by otter scat collected at latrines often spaced kilometers apart. Meanwhile, the significant differences in contaminant concentrations between geographic zones suggest that this uncoupling does not span longer stretches of the river, despite evidence of at least occasional travel of otters between zones (two of six repeat detections, 25 and 32 km apart).

### River otters as biomonitors

The urbanization gradient of the Green-Duwamish River and its concomitant variation in environmental quality provide a valuable opportunity to test the utility of river otters as biomonitors. Otters are integrating contaminant inputs to the river system from multiple sources, including differential loading along the river associated with historical and current patterns of land use in each of the development zones, as well as pollutants carried downstream from upstream sources. The strong predictive power of development zone indicates that river otters are effective biomonitors at the regional scale defined in this study by impervious surface land cover (Fig. 1), which is a key indicator of urbanization and its resultant hydrological, physical, and ecological impacts, including pollution (Arnold & Gibbons, 1996). This is corroborated by assessments of water quality, sediment, and biota, which demonstrate similar patterns of increasing contaminant concentrations with distance downstream and with increasing urbanization (Ecology & King County, 2011; King County, 2018; O’Neill et al., 2015), with a peak of contamination leading to USEPA Superfund designation in what this study characterized as the industrial zone. Although the mobility of otters and their prey could introduce confounding factors for river otters as biomonitors at the latrine scale, otters are suitable at the regional scale evaluated in this study. Though our genetic-recapture sample size was limited, four of six repeat detections of individuals were in the same geographic zones at latrines a maximum of 5.5 km apart, suggesting a range relevant to discriminating our geographic zones. Core river otter ranges of less than 5 shoreline km or 20 km^2^ have been documented in other studies (e.g., Erickson et al., 1984; Gorman et al., 2006; Helon et al., 2004; Melquist & Hornocker, 1983). Meanwhile, a corollary study of prey hard parts in otter scat samples showed that across all geographic zones, 79% of prey items were considered resident (less-mobile) species (C. Straight, unpublished data), indicating that prey mobility is unlikely to compromise river otters as biomonitors, at least during the months of our study. However, these patterns may change during significant runs of the salmonid species native to the river. A more detailed assessment is needed to determine whether spikes in migratory salmonid predation would weaken the efficacy of river otters as biomonitors during these particular events. In addition, investigation of seasonal and annual comparisons, which were precluded in our study due to logistical and sample size constraints, would allow for valuable assessments of the temporal scales reflected by contaminant concentrations in river otter scat.

Extensive study of river otters along the south coast of Vancouver Island, British Columbia, Canada, has revealed similar spatial contaminant patterns as those reported in our study, with individuals in federally-designated contaminated harbors experiencing higher exposure to pollutants (PCBs, PBDEs, and organochlorine pesticides) relative to those inhabiting the more natural setting of adjacent shorelines (Guertin et al., 2010, 2012; Huang et al., 2018; Nelson et al., 2015). Spatial patterns of anthropogenic impacts have also been reflected by river otter in other North American and European sites. Concentrations of three out of four flame retardants evaluated along the tri-river system of Missoula, MT, USA, were positively correlated with human population density (La Guardia et al., 2020), while mean ΣPCB levels in river otter liver and scat samples were highest in the most populated and industrial areas of Oregon and Washington (Grove & Henny, 2008; Henny et al., 1981, 1996). Differences in river otter fecal porphyrin levels were also indicative of oiled and non-oiled sites in Prince Williams Sound, Alaska, USA, after the *Exxon Valdez* oil spill (Bowyer et al., 2003). Decades of research in Europe reveal alignment of pollutant burdens in Eurasian otters (*Lutra lutra*) with anthropogenic environmental pressures (Christensen et al., 2010; Delibes et al., 2009; Lemarchand et al., 2007; Mason et al., 1992; O’Sullivan et al., 1993; Poutney et al., 2015; Roos et al., 2001). Similar concordance between river otter pollutant burdens and environmental contamination has also been documented on a temporal scale, with significant declines in otter tissue concentrations over decades as pollutants have been phased out or banned (Bowyer et al., 2003; Elliott et al., 1999; Grove & Henny, 2008; Mason, 1998; Roos et al., 2001, 2012).

Many authors have debated the utility of river otters as sentinels, bioindicators or biomonitors, but these terms have carried varying definitions and as such have led to conflicting conclusions (Bowyer et al., 2003; Carpenter et al., 2014; Delibes et al., 2009; Peterson & Schulte, 2016; Reid et al., 2013; Ruiz-Olmo et al., 2000). Based on the strengths and limitations of this study, we conclude that the river otter is an excellent biomonitor, precisely defined, of environmental and food web contaminant exposure at the scale of our development zones in the Green-Duwamish River. As a piscivore but also opportunistic predator, we consider the river otter an organism that is a good integrator of the aquatic food web and a useful species for determining environmental, wildlife, and human contaminant exposure, consistent with Grove et al. (2001). Specifically, river otters and their scat contain information on quantitative aspects of the quality of their environment (Markert et al., 2003)—in this case, contaminant levels in the local food web and environment of the Green-Duwamish River. It is unclear whether their consistent presence represents a lack of sensitivity to their contaminant burden, “bad decisions” about habitat use (Delibes et al., 2009), and/or underlying source-sink population dynamics. However, in this setting, it is apparent that river otters do not appear to be “canaries in the coal mine” that disappear in degraded habitats, or conversely, that their presence indicates high quality habitat.

The USEPA released their Record of Decision detailing restoration and remediation plans for the Lower Duwamish Waterway Superfund site in 2014 (USEPA, 2014) and negotiations regarding implementation and monitoring of the estimated 17-year effort are currently underway. Baseline sampling of sediment, water, fish, crab, and clam was conducted in 2017 and 2018 (Windward, 2020). Our data (and an expanded sample size in Leidos & Wainstein, 2017) represent the only empirically based index of contaminants in an apex predator or mammal in this system and a convincing integrated measure of the ecological footprint of pollutants in the local food web and environment. This study provides baseline data contemporaneous to the aforementioned studies, as well as upstream data that can serve as benchmarks for effectiveness monitoring of restoration efforts. Scat samples can be collected non-invasively, easily and affordably in the terrestrial environment, making river otter a key species for long-term monitoring and future assessments of the ecological impacts of remediation and restoration efforts in the Lower Duwamish Waterway.

### Contaminant levels and potential for health impacts

#### PCBs

While Σ_40_PCB concentration levels were high in at least some river otter scat samples from both the industrial and suburban development zones, those collected in the Lower Duwamish Waterway (LDW), a US federally designated contamination site within the Green-Duwamish River, reflect Σ_40_PCB exposure among the highest published levels in a wild river otter population (Table 1). The Σ_40_PCBs were nearly always the highest concentration contaminant in the LDW (our industrial geographic zone) and they reflect a legacy of contamination. Pollutants entered this section of river for decades through spills, leaks, dumping, and inappropriate management practices (USEPA, 2014) and were then trapped by man-made modifications of the estuary that decreased current velocities and increased sediment deposition (Windward, 2007). The geometric mean (14 mg/kg lw) of Σ_40_PCBs in otter scat from the Lower Duwamish Waterway (industrial zone) is to our knowledge the highest published to date, and similar to the comparable federally-designated contamination sites of Victoria and Esquimalt Harbors in British Columbia, Canada (highest means: 12 mg/kg lw in Elliott et al., 2008; 11 mg/kg lw in Guertin et al., 2010; 5.5 mg/kg lw in Huang et al., 2018). Elliott et al. (2008) compiled mean ΣPCB concentrations in river otter scat from twenty different publications reporting on over 60 locales from mostly European regions. While they note that comparisons should be made with caution given variable approaches to sampling, laboratory procedures (especially the number of congeners), and statistical analyses, they are valuable for broad comparative purposes. Our industrial zone geometric mean Σ_40_PCB concentration (14 mg/kg lw, a conservative value compared to most means in the table, which are arithmetic) exceeds all but three of the 69 reported means. Two (24 and 36 mg/kg lw) were recorded in the Lower-Saxony region of Germany where otter populations had experienced dramatic declines (Reuther & Mason, 1992 as cited in Elliott et al., 2008) and the third (19 mg/kg lw) was reported from the Lower River Clyde of Scotland, a grossly polluted area in which the authors presumed river otters could only be transient (Mason et al., 1992). Our maximum individual scat Σ_40_PCB concentration (180 mg/kg lw) exceeded the maximum in British Columbia, Canada (108 mg/kg; Elliott et al., 2008) and matched the maximum concentration from the Lower River Clyde of Scotland (180 mg/kg lw, Mason et al., 1992). Of the 63 maximum ΣPCB values collated by Elliott et al. (2008), no other exceeded 107 mg/kg lw and 92% of maximum values were below 70 mg/kg lw.

The scope of the current study did not allow for direct assessment of health impacts of contaminants on otters of the Green-Duwamish River. However, published toxicological thresholds and other contaminant studies provide the opportunity to discuss the potential for detrimental effects. Fecal ΣPCB concentration reference points for river otters (*L. lutra* and *L. canadensis*) were developed in a two-step model based primarily on closely related American mink (*Mustela vison*) dosing experiments and have been applied widely (e.g., Elliott et al., 2008; Guertin et al., 2010; Huang et al., 2018; Kruuk & Conroy, 1996; Mason & Macdonald, 1994; Mason et al., 1992). Forty-six percent of our industrial zone scat samples suggest exposures above putative ΣPCB critical levels (16 mg/kg lw), while almost another quarter of scat analyzed in our study exceeded the level of concern (9–16 mg/kg lw; Fig. 4). This total of almost 70% of our samples is 2.5 times the percentage of scat samples that exceeded the level of concern in Victoria Harbor, Canada (Huang et al., 2018), though percentages reached 55% in the past (Guertin et al., 2010). In Western Britain, where contaminants are believed to have driven severe population declines, 23% of otter scat samples exceeded the critical level and another 47% exceeded the level of concern in regions with the highest human, industrial, and sewage discharge activity (Mason & Macdonald, 1993b). Numerous other studies in Europe describe otter populations exposed above the critical threshold, though percentages do not generally exceed roughly 25% (Mason & Macdonald, 1994; O’Sullivan et al., 1993; see also Ruiz-Olmo et al., 2000 for a review of multiple studies using the corresponding critical tissue levels).

Primarily correlational evidence has been accumulating for the likely detrimental impacts of PCBs on individual wild river otters, including increased frequency of disease (Leonards et al., 1996), decreased body condition and vitamin A levels (Kruuk & Conroy, 1996; Murk et al., 1998; Simpson et al., 2000), increased bone pathologies (Roos et al., 2010), and reproductive disorders and gross organ abnormalities (Henny et al., 1996). Huang et al. (2018) showed disruptions in triiodothyronine (T_3_ thyroid hormone) and progesterone in Canadian river otters at PCB levels below our reported concentrations, perturbations that could adversely affect homeostatic processes and the reproductive cycle of females. Several studies measured multiple contaminant classes with co-varying concentrations, making it challenging to discriminate between these classes relative to specific toxicological effects (e.g., Henny et al., 1996; Huang et al., 2018).

The USEPA Baseline Ecological Risk Assessment for the Lower Duwamish Waterway Superfund site specifically identified the river otter as a species of concern and evaluated lowest-observed-adverse-effect-level (LOAEL) hazard quotients to quantify the risk of adverse effects from contaminants. This approach was entirely model-based and there were no data from local otters included in the calculations (Windward, 2007). The assessment calculated a hazard quotient (dietary dose:adverse dose) of 2.9, suggesting that estimated dietary doses of ΣPCBs were nearly three times greater than the LOAEL doses presumed to cause adverse effects in river otters. Taking a comparable approach, using our observed geometric mean ΣPCB concentrations and the level of concern threshold value (9 mg/kg lw) developed for otter scat to generate a similar ratio, we find hazard quotients among the six latrines in the industrial zone ranging from 1 to 6.7, with an overall quotient of 1.6 for all scat samples in the Lower Duwamish Waterway. At the time of our study, remediation of targeted “Early Action Areas”—portions of the river with the most contaminated sediment—had already been completed, which helped reduce PCB sediment concentrations in the waterway by approximately half (USEPA, 2019). This may help explain our lower overall hazard quotient; meanwhile, it may also suggest that until recently, river otters in the LDW may have been exposed to considerably higher levels of PCBs. Additionally, very high risk quotients at some individual latrines also indicate continued exposure to potentially harmful levels of PCBs.

#### PBDEs

Though PBDEs have been evaluated much less in wildlife than PCBs, Σ_11_PBDE river otter scat concentrations in the industrial zone of the Green-Duwamish River (geometric mean = 0.45 mg/kg lw with one relatively high value of 2.2 mg/kg lw; Table 1) appear similar to comparable reports elsewhere. Two studies of river otter scat from the polluted Victoria Harbor of Vancouver Island, Canada, revealed geometric mean concentrations of Σ_12_PBDE = 0.36 and Σ_20_PBDE = 0.35 mg/kg lw (Guertin et al., 2010; Nelson et al., 2015). Guertin et al. (2010) also reported a maximum Σ_12_PBDE value of 2.7 mg/kg lw and Nelson et al. (2015) detected two samples with extremely high levels of BDE-209. Meanwhile, La Guardia et al. (2020) reported a maximum mean Σ_9_PBDE concentration of 0.41 mg/kg lw, comparable to our mean, in otter scat from areas downstream of a large urban center in a river system in Montana, USA. Conversions to liver tissue concentration estimates for comparisons to toxicological studies as in La Guardia et al. (2020) indicate Σ_9_PBDE levels far below the NOAEL for juvenile mink (Zhang et al., 2009) in all but one scat sample (Table S7), suggesting that the PBDEs measured, at least in isolation, are unlikely to compromise the health of otters in the Green-Duwamish River.

#### Organochlorine pesticides

Organochlorine (OC) pesticide concentrations in river otters have been reported in varied combinations, and our geometric means were comparable for many pesticide compounds and categories that overlapped with similar studies. For example, our industrial zone geometric mean of a comparable Σ_17_OCs (supplementary information, Table S8) including HCB, Σ_3_HCHs, Σ_8_CHLDs, Σ_3_DDTs, dieldrin, and mirex was 0.69 mg/kg lw, while means reported for river otters in polluted Victoria Harbor, Canada ranged from 0.37 to 0.70 mg/kg lw (Elliott et al., 2008; Guertin et al., 2010; Huang et al., 2018). Compared to DDE levels measured in pooled river otter scats from the Lower Columbia River (OR/WA, USA; Henny et al., 1996), our mean industrial zone Σ_6_DDT concentration was approximately an order of magnitude lower, though some of our maximum values were comparable (e.g., scats at DIA, HAM, and KCO; Table S8). Our OC levels were also notably lower than mean OC concentrations in several studies of Eurasian otters (*Lutra lutra*), which were considered low relative to other contaminants detected (Lemarchand et al., 2007; Mason & Macdonald, 1993b; Mason et al., 1992; O’Sullivan et al., 1993). These Canadian and European studies conclude that OCs are unlikely to impact river otters, especially compared to the PCB levels observed in those otter populations. Our comparatively low levels of OCs, plus Σ_40_PCB and Σ_37_PAH concentrations roughly 50 and 40 times that of Σ_17_OCs, respectively, suggest that organochlorides in the Green-Duwamish River are unlikely to contribute significantly to adverse impacts on otters.

#### Diet factors

Across all POPs, river otter diet may be amplifying exposure to contaminants, particularly in the polluted Lower Duwamish Waterway (industrial zone). Lipid content of scat samples, and therefore prey items, was significantly higher in the industrial zone, and the majority of contaminants we investigated are lipophilic (Jones & de Voogt, 1999). Though otter diet data are limited for this region, predation on higher lipid-content species would increase intake of the lipophilic POPs we measured. Additionally, the majority (91%) of our scat samples from the LDW contained primarily fish hard parts, corroborated by the high δ^15^N values for these samples as compared to lower δ^15^N values for crustacean-dominated or mixed-prey scats (Fig. S1). Because POPs biomagnify in successive trophic levels (Elliott et al., 2009; Meador et al., 2010), this fish-dominated diet in the LDW also serves to concentrate contaminants in otters. In an analysis of hard parts in scat samples collected as a corollary to this study, the most common prey items detected were sculpins (Cottidae; C. Straight, unpublished data), which have among the highest PCB concentrations of fish sampled in the LDW (Windward, 2010). Furthermore, over 96% of prey species in industrial zone scats from this corollary work were identified as either resident, bottom-feeding or both, characteristics that may lead to an increased likelihood of accumulating contaminants. Because of their high concentration of contaminants, several of the high-frequency prey items from river otter scat are also identified as resident species to avoid in the Duwamish River human health advisories circulated by the Washington State Department of Health (WADOH, 2014). The key role of diet factors in influencing exposure to pollutants is further illustrated by the importance of both δ^15^N and lipid content in predictive regression models for all the contaminants we measured.

#### PAHs

The Σ_37_PAH concentrations often approached, and at one latrine exceeded, Σ_40_PCBs in river otter scat samples in the industrial zone, with a geometric mean of 11 mg/kg lw (Table 1). Meanwhile, Σ_37_PAH levels consistently exceeded Σ_40_PCBs in the suburban and rural zones. As with other contaminant categories described above, there are limited comparable studies of the potential impacts of PAHs on free-ranging mammalian wildlife, and we know of no published studies with comparable species, sampling matrices, laboratory procedures, and analytical methods. Livers from river otters collected in Alberta, Canada, which were exposed to PAH levels almost two orders of magnitude (Σ_46_PAH < 0.14 mg/kg lw) lower than those found in our study, showed disruptions of stress and reproductive hormones, and negative effects on baculum bone material properties, which could impact reproductive success (Thomas, 2020; Thomas et al., 2021). Sea otters (*Enhyrda lutris*) from British Columbia, Canada and Washington and California, USA, had liver and blood wet weight PAH concentrations mostly well below our river otter scat, some by an order of magnitude (Brancato et al., 2009; Harris et al., 2011; Kannan & Perrotta, 2008). However, interpretation remains difficult for these cross-tissue comparisons because PAHs, unlike many POPs, can be rapidly metabolized in vertebrates to more polar compounds that are readily excreted (Meador et al., 1995; Thomas, 2020; Varanasi et al., 1993; Ylitalo et al., 2017). A study using fecal samples from Southern Resident Killer Whales (SRKW, *Orcinus orca*) near San Juan Island, WA, reported a Σ_25_PAH mean concentration of less than 10 ng/g ww (Lundin et al., 2018) compared to our geometric mean in the industrial zone of 93 ng/g ww (Table 1), though they tested notably fewer analytes. Even outlier values for SRKW ranging as high as 104 ng/g ww during presumed increased exposure to combustion engine emissions (Lundin et al., 2018) were comparable to our mean and at least an order of magnitude below our highest industrial levels. The relatively high levels of Σ_37_PAHs in river otter scat, considering also the exclusion of naphthalenes in our totals, suggest potential consequences for river otters throughout the Green-Duwamish system.

Despite these likely adverse impacts from a diverse array of environmental contaminants on individual otters (a legitimate animal welfare issue of its own), density-dependent compensatory reproduction and mortality may attenuate any relationship with population dynamics (Newton, 1988). Population-level impacts have been widely debated, especially as contaminants relate to declines and recoveries of Eurasian otter populations in Europe (e.g., Mason, 1997 vs Kruuk, 1997; see also Lammertsma & van den Brink, 2012 and Smit et al., 1998). In North America, detailed population genetics approaches have allowed exploration of the relationship between toxicity and population health by evaluating source-sink population dynamics relative to contaminant loads in individual river otters (Huang et al., 2018; Thomas, 2020; see also Bowyer et al., 2003). There have been no population-level studies in the Green-Duwamish River, though there is anecdotal evidence from residents and tradespeople that otters, including females with kits (which do not travel far from natal dens), have frequented the river for decades, even in the Superfund zone (M. Wainstein, personal observation). Our data demonstrate that highly contaminated river otters are persisting in the Lower Duwamish Waterway. Because the toxicity thresholds established for otters were partially based on toxicity experiments conducted on mink, this persistence of otters despite contaminant levels exceeding the thresholds suggests that otters may not be as sensitive to contaminants as mink. This has been proposed by other studies where otters are present or even thriving despite high pollutant burdens (Grove & Henny, 2008; Guertin et al., 2012; Huang et al., 2018; Kruuk & Conroy, 1996). While our scat contaminant concentrations suggest remarkably high pollutant levels in otters of the Lower Duwamish Waterway, especially for PCBs and PAHs, understanding individual and population health impacts would require considerable additional research, especially given the potential for additive effects that are difficult to measure in the wild and not represented by controlled experimental studies on individual contaminant classes.

## Conclusions

We report concentrations of PCBs, PAHs, PBDEs, DDTs, CHLDs, and HCB in river otters along the extreme urbanization gradient of the Green-Duwamish River, and demonstrate that industrial, suburban, and rural geographic zones are strong predictors of contaminant concentration patterns. Pollutant levels in river otter scat are reflective of regional abiotic and biotic conditions and therefore otters represent an excellent biomonitor of food web and environmental contaminant exposure in this river system. We recommend that river otter scat be included in evaluating the broader ecological impacts of long-term remediation and restoration efforts in the Lower Duwamish Waterway, and suggest it may be a useful monitoring tool wherever river otters are found. Furthermore, we report some of the highest concentrations of PCBs and PAHs published to date, and evidence of all other contaminants measured, in river otter scat from the Lower Duwamish Waterway, a highly polluted urban estuary. While PCBs in particular exceeded published toxicological effects thresholds, the scope of our study precluded evaluation of individual or population effects of pollutant burdens.

## Supplementary information

Below is the link to the electronic supplementary material.

Supplementary file1 (DOCX 135 KB)

Supplementary file2 (XLSX 80.1 KB)

## Data Availability

The data that support the findings of this study are available in the Supplementary Data File provided online in association with this article.

## Code availability

N/A.
