# Supplementary material for: Highly contaminated river otters (Lontra canadensis) are effective biomonitors of environmental pollutant exposure
Source: Environ Monit Assess. 2022 Aug 16;194(10):670. doi: 10.1007/s10661-022-10272-9 (PMC9378324; doi:10.1007/s10661-022-10272-9)
Supplement: Supplementary file 1 — Supplementary file1 (DOCX 135 KB) [file 10661_2022_10272_MOESM1_ESM.docx]

**SUPPLEMENTARY INFORMATION**

**Highly contaminated river otters (*Lontra canadensis*) are effective biomonitors of environmental pollutant exposure**

**Author Information**

Michelle Wainstein^a^*, Louisa B. Harding^b^, Sandra M. O'Neill^b^, Daryle T. Boyd^c^, Fred Koontz ^d^, Bobbi Miller^e^, Cornelya F. C. Klütsch^f^, Philippe J. Thomas^g^, Gina M. Ylitalo^c^

^a^ Conservation, Research and Education Opportunities, Seattle, WA 98107, USA

^b^ Washington Department of Fish and Wildlife, PO Box 43200, Olympia, WA 98504-3200, USA
^c^ Northwest Fisheries Science Center, National Marine Fisheries Service, National Oceanic and Atmospheric Administration, 2725 Montlake Boulevard East, Seattle, WA 98112-2097, USA

^d^ Duvall, WA 98019, USA

^e^ Woodland Park Zoo, 5500 Phinney Ave N, Seattle, WA 98103, USA

^f^ Norwegian Institute of Bioeconomy Research (NIBIO), Division of Environmental Research in the Barents Region, NIBIO Svanhovd, Svanhovd, NO-9925 Svanvik, Norway

^g^ Science and Technology Branch, Environment and Climate Change Canada, National Wildlife Research Center, 1125 Colonel By Drive, Raven Road, Ottawa, ON, K1A 0H3, Canada

* corresponding author: michelle@creoi.org

**Contents**

**Table S1.** Thirty-seven PAH analytes measured in river otters scat samples collected along the Green-Duwamish River, WA

**Table S2.** Arithmetic mean concentrations and standard deviations (±SD) of Σ_40_PCB, Σ_11_PBDE, Σ_6_DDT, and Σ_37_PAH in river otter (*Lontra canadensis*) scat collected from 12 latrines in three development zones along the Green-Duwamish River in Washington State, USA

**Table S3.** Geometric mean concentrations and ranges, and arithmetic mean concentrations and standard deviations (±SD) of Σ_8_CHLD and HCB in river otter (*Lontra canadensis*) scat collected from 12 latrines in three development zones along the Green-Duwamish River in Washington State, USA

**Table S4.** Comparison of linear models assessing the effects of development zone (DevZone) or latrine site, δ^15^N, and scat lipid content (natural log-transformed percent lipid) on ∑_40_PCB, ∑_11_PBDE, ∑_6_DDT, and ∑_42_PAH concentrations in river otter (*Lontra canadensis*) scat samples

**Table S5.** Predicted mean concentrations, standard errors (SE), and 95% confidence intervals (CI) for the best-fitting model for ∑_6_DDTs (ln values) using the mean lipid content (ln values) in each of three development zones (grand mean results presented in Table 2) along the Green-Duwamish River, Washington State, USA

**Table S6.** Predicted mean concentrations, standard errors (SE), and 95% confidence intervals (CI) for percent lipid, and δ^15^N in river otter (*Lontra canadensis*) scat samples from three development zones along the Green-Duwamish River in Washington State, USA

**Table S7.** Arithmetic means and ranges of ∑_11_PBDE concentrations (mg/kg lw) in river otter (*Lontra canadensis*) scat collected from 12 latrines in three development zones along the Green-Duwamish River in Washington State, USA, and their conversions to ∑_11_PBDE liver tissue concentration (mg/kg lw) estimates following La Guardia et al. (2020) body burden estimates

**Table S8.** Geometric mean concentrations and ranges of Σ_17_OCs in river otter (*Lontra canadensis*) scat collected from 12 latrines in three development zones along the Green-Duwamish River in Washington State, USA

**Figure S1.** δ^15^N in river otter scat compared to gross field characterization of hard parts present in otter scat during sample collection along the Green-Duwamish River, WA

**Genetics detail**

Methods

Results

**Table S9.** Number of alleles (NA), allelic range (A_R_), observed heterozygosity (H_O_), expected (H_E_), and F_IS_ plus p-values for microsatellite loci amplified from 18 unique river otters collected along the Green-Duwamish River, WA, USA.

**Table S10.** A matrix of relatedness for 18 unique river otters identified from microsatellite DNA analysis of scat collected along the Green-Duwamish River, WA. For each individual, the three letter code (e.g., CWG) represents the latrine from which the scat was collected (see Fig. 1) and the number represents the scat sample. U = unrelated; HS = half sibling; FS = full sibling; PO = parent-offspring.

References

**Table S1.** Thirty-seven PAH analytes measured in river otters scat samples collected along the Green-Duwamish River, WA. LMW = low molecular weight; HMW = high molecular weight. Five naphthalene analytes (NPHs = NPH, C_1_NPH, C_2_NPH, C_3_NPH, C_4_NPH) were excluded from analyses because laboratory blanks contained NPHs at high enough concentrations to account for observed levels in scat samples.

| LMW analytes |  | HMW analytes |
| --- | --- | --- |
| Acenaphthylene (ACY) |  | Fluoranthene (FLA) |
| Acenaphthene (ACE) |  | Pyrene (PYR) |
| Fluorene (FLU) |  | C_1_FLA/PYR |
| C_1_FLU |  | C_2_FLA/PYR |
| C_2_FLU |  | C_3_FLA/PYR |
| C_3_FLU |  | C_4_FLA/PYR |
| Dibenzothiophene (DBT) |  | Benz[*a*]anthracene (BAA) |
| C_1_DBT |  | Chrysene (CHR)^a^ |
| C_2_DBT |  | C_1_CHR/BAA |
| C_3_DBT |  | C_2_CHR/BAA |
| C_4_DBT |  | C_3_CHR/BAA |
| Phenanthrene (PHN) |  | C_4_CHR/BAA |
| Anthracene (ANT) |  | Benzo[*b*]fluoranthene (BBF) |
| C_1_PHN/ANT |  | Benzo[*k*]fluoranthene (BKF)^b^ |
| C_2_PHN/ANT |  | Benzo[*e*]pyrene (BEP) |
| C_3_PHN/ANT |  | Benzo[*a*]pyrene (BAP) |
| C_4_PHN/ANT |  | Perylene (PER) |
|  |  | Dibenz[*a,h*]anthracene (DBA)^c^ |
|  |  | Indeno[*1,2,3-cd*]pyrene (IDP) |
|  |  | Benzo[*ghi*]perylene (BZP) |
|  |  |  |

^a^  Coeluted with triphenylene.

^b^  Coeluted with benzo[*j*]fluoranthene.

^c^  Coeluted with dibenz[*a,c*]anthracene.

**Table S2.** Arithmetic mean concentrations and standard deviations (±SD) of Σ_40_PCB, Σ_11_PBDE, Σ_6_DDT, and Σ_37_PAH in river otter (*Lontra canadensis*) scat collected from 12 latrines in three development zones along the Green-Duwamish River in Washington State, USA. Mean concentrations are reported as ng/g wet weight (ww) and mg/kg lipid weight (lw). Development zones are based on impervious surface land cover as defined in the methods. Maximum latrine and development zone contaminant concentrations are indicated in bold. River KM, percent lipids (Lipids), and δ^15^N are presented in Table 1.

| **Development Zone** | **Latrine** | **N** | **Σ_40_PCB** | |  | **Σ_11_PBDE** | |  | **Σ_6_DDT** | |  | **Σ_42_PAH** | |
| --- | --- | --- | --- | --- | --- | --- | --- | --- | --- | --- | --- | --- | --- |
|  |  |  | **ww** | **lw** |  | **ww** | **lw** |  | **ww** | **lw** |  | **ww** | **lw** |
| Industrial | HIM | 3 | 150 | 12 |  | 3.9 | 0.32 |  | 2.6 | 0.20 |  | 130 | 9.7 |
|  |  |  | (91-270) | (4.7-24) |  | (1.3-9.0) | (0.071-0.80) |  | (1.2-5.1) | (0.071-0.45) |  | (110-160) | (5.2-12) |
|  | DIA | 2 | **740** | **100** |  | **9.4** | **1.3** |  | 5.8 | **0.82** |  | 310 | 71 |
|  |  |  | (84-**1400**) | (20-**180**) |  | (1.8-**17**) | (0.42-**2.2**) |  | (1.5-10) | (0.35-1.3) |  | (22-600) | (2.8-140) |
|  | SPM | 12 | 150 | 17 |  | 4.9 | 0.50 |  | 2.8 | 0.26 |  | **530** | 23 |
|  |  |  | (17-310) | (5.5-46) |  | (0.60-15) | (0.27-0.97) |  | (0.15-**12**) | (0.057-0.83) |  | (8.0-**4400**) | (2.5-80) |
|  | BOE | 2 | 110 | 24 |  | 2.8 | 0.62 |  | 2.2 | 0.50 |  | 240 | **78** |
|  |  |  | (63-160) | (21-27) |  | (1.9-3.6) | (0.60-0.64) |  | (1.6-2.7) | (0.45-0.54) |  | (39-450) | (6.5-**150**) |
|  | HAM | 8 | 130 | 16 |  | 3.9 | 0.49 |  | 2.7 | 0.44 |  | 440 | 25 |
|  |  |  | (31-400) | (2.8-43) |  | (0.96-9.4) | (0.086-1.0) |  | (0.91-4.5) | (0.076-1.7) |  | (13-2500) | (0.61-120) |
|  | KCO | 8 | 150 | 18 |  | 6.8 | 0.73 |  | **6.2** | 0.73 |  | 180 | 12 |
|  |  |  | (62-340) | (3.1-48) |  | (2.6-**17**) | (0.074-1.2) |  | (2.5-11) | (0.099-1.5) |  | (16-1100) | (0.45-56) |
|  | All industrial | 35 | **180** | **22** |  | **5.2** | **0.59** |  | **3.7** | **0.45** |  | **360** | **26** |
|  |  |  | (17-**1400**) | (2.8-**180**) |  | (0.60**-17**) | (0.071-**2.2**) |  | (0.15-**12**) | (0.057-1.7) |  | (8-**4400**) | (0.45-**150**) |
| Suburban | FGL | 7 | 15 | 2.9 |  | 2.5 | 0.45 |  | 1.3 | 0.23 |  | 27 | 5.5 |
|  |  |  | (5.9-31) | (1.2-4.4) |  | (0.27-7.2) | (0.10-1.0) |  | (0.082-4.3) | 0.029-0.84) |  | (5.1-79) | (0.95-11) |
|  | BLK | 7 | 24 | 4.5 |  | 2.8 | 0.50 |  | 1.3 | 0.16 |  | 47 | 9.4 |
|  |  |  | (10-33) | (1.9-9.4) |  | (0.73-4.5) | (0.18-0.87) |  | (0.22-4.6) | (0.072-0.31) |  | (25-120) | (2.1-21) |
|  | CWG | 7 | 19 | 3.1 |  | 2.3 | 0.37 |  | 3.7 | 0.58 |  | 18* | 3.6* |
|  |  |  | (2.0-71) | (0.91-11) |  | (0.17-8.4) | (0.078-1.3) |  | (0.16-11) | (0.073-**1.9**) |  | (1.2-43) | (0.41-7.5) |
|  | All suburban | 21 | 19 | 3.5 |  | 2.6 | 0.44 |  | 2.1 | 0.33 |  | 31* | 6.1* |
|  |  |  | (2.0-71) | (0.91-11) |  | (0.17-8.4) | (0.078-1.3) |  | (0.082-11) | (0.029-**1.9**) |  | (1.2-120) | (0.41-21) |
| Rural | GNA | 4 | 1.3 | 0.56 |  | 0.14* | 0.053* |  | 0.10* | 0.040* |  | 4.3 | 1.4 |
|  |  |  | (0.71-1.6) | (0.25-1.3) |  | (0.098-0.19) | (0.031-0.078) |  | (0.080-0.16) | (0.019-0.063) |  | (2.0-9.6) | (0.54-2.3) |
|  | ICY | 3 | 1.3 | 0.63 |  | 0.13* | 0.056* |  | 0.12* | 0.051* |  | 5.1 | 2.5 |
|  |  |  | (0.84-2.0) | (0.17-0.99) |  | (0.070-0.21) | (0.022-0.10) |  | (0.070-0.16) | (0.032-0.074) |  | (1.0-13) | (0.20-6.4) |
|  | LIR | 6 | 6.3 | 0.73 |  | 0.65* | 0.082* |  | 1.0* | 0.071* |  | 8.4 | 1.1 |
|  |  |  | (1.0-20) | (0.42-1.4) |  | (0.13-1.8) | (0.041-0.18) |  | (0.070-5.1) | (0.029-0.16) |  | (2.7-28) | (0.88-1.4) |
|  | All rural | 13 | 3.6 | 0.65 |  | 0.37* | 0.067* |  | 0.54* | 0.057* |  | 6.4 | 1.5 |
|  |  |  | (0.71-20) | (0.17-1.4) |  | (0.070-1.8) | (0.022-0.18*)* |  | (0.070-5.1) | (0.019-0.16) |  | (1.0-28) | (0.20-6.4) |

Five naphthalene analytes (NPHs = NPH, C_1_NPH, C_2_NPH, C_3_NPH, C_4_NPH) were excluded from analyses because laboratory blanks contained NPHs at high enough concentrations to account for observed levels in scat samples.

*****Mean and associated range include at least one less than the lower limit of quantitation (<LOQ) replacement value randomly generated from the range of <LOQs for the relevant contaminant category.

**Table S3.** Geometric mean concentrations and ranges, and arithmetic mean concentrations and standard deviations (±SD) of Σ_8_CHLD and HCB in river otter (*Lontra canadensis*) scat collected from 12 latrines in three development zones along the Green-Duwamish River in Washington State, USA. Mean concentrations are reported as ng/g wet weight (ww) and mg/kg lipid weight (lw). Development zones are based on impervious surface land cover as defined in the methods. Maximum latrine and development zone contaminant concentrations are indicated in bold. River KM, means of ln-transformed percent lipids (Lipids), and δ^15^N are presented in Table 1.

| **Development Zone** | **Latrine** | **N** | **GEOMETRIC MEANS** | | | | |  | **ARITHMETIC MEANS** | | | | |
| --- | --- | --- | --- | --- | --- | --- | --- | --- | --- | --- | --- | --- | --- |
|  |  |  | **Σ_8_CHLD** | |  | **HCB** | |  | **Σ_8_CHLD** | |  | **HCB** | |
|  |  |  | **ww** | **lw** |  | **ww** | **lw** |  | **ww** | **lw** |  | **ww** | **lw** |
| Industrial | HIM | 3 | 1.5 | 0.10 |  | 0.42 | 0.028 |  | 1.6 | 0.12 |  | 0.45 | 0.029 |
|  |  |  | (1.1-2.1) | (0.076-0.19) |  | (0.24-0.61) | (0.021-0.036) |  | (1.1-2.1) | (0.076-0.19) |  | (0.24-0.61) | (0.021-0.036) |
|  | DIA | 2 | 1.1 | 0.20 |  | 0.26* | 0.045* |  | 2.5 | 0.34 |  | 0.30* | 0.046* |
|  |  |  | (0.28-4.7) | (0.065-0.61) |  | (0.15-0.45) | (0.035-0.058) |  | (0.28-4.7) | (0.065-0.61) |  | (0.15-0.45) | (0.035-0.058) |
|  | SPM | 12 | 1.5 | 0.20 |  | 0.36* | 0.047* |  | 2.4 | 0.22 |  | 0.54* | 0.050* |
|  |  |  | (0.14-8.1) | (0.099-0.44) |  | (0.090-**2.4**) | (0.025-0.085) |  | (0.14-8.1) | (0.099-0.44) |  | (0.090-**2.4**) | (0.025-0.085) |
|  | BOE | 2 | 1.1 | 0.25 |  | 0.24 | 0.056 |  | 1.1 | 0.25 |  | 0.24 | 0.056 |
|  |  |  | (0.69-1.6) | (0.23-0.27) |  | (0.18-0.31) | (0.052-0.060) |  | (0.69-1.6) | (0.23-0.27) |  | (0.18-0.31) | (0.052-0.060) |
|  | HAM | 8 | 2.1 | 0.24 |  | 0.79 | 0.090 |  | 2.4 | 0.40 |  | 0.85 | 0.10 |
|  |  |  | (0.79-4.2) | (0.057-1.1) |  | (0.35-1.3) | (0.041-0.19) |  | (0.79-4.2) | (0.057-1.1) |  | (0.35-1.3) | (0.041-0.19) |
|  | KCO | 8 | **7.3** | **0.73** |  | **0.89** | 0.090 |  | **11** | **1.2** |  | **0.94** | 0.10 |
|  |  |  | (2.0-**30**) | (0.12-**4.2**) |  | (0.44-1.4) | (0.040-**0.23**) |  | (2.0-**30**) | (0.12-**4.2**) |  | (0.44-1.4) | (0.040-**0.23**) |
|  | All industrial | 35 | **2.3** | **0.27** |  | **0.51*** | 0.061* |  | **4.1** | **0.49** |  | **0.66*** | 0.073* |
|  |  |  | (0.14-**30**) | (0.057-**4.2)** |  | (0.090-**2.4**) | (0.021-**0.23**) |  | (0.14-**30**) | (0.057-**4.2)** |  | (0.090-**2.4**) | (0.021-**0.23**) |
| Suburban | FGL | 7 | 0.71 | 0.16 |  | 0.21* | 0.046* |  | 1.4 | 0.22 |  | 0.23* | 0.047* |
|  |  |  | (0.057-3.8) | (0.021-0.44) |  | (0.12-0.41) | (0.025-0.060) |  | (0.057-3.8) | (0.021-0.44) |  | (0.12-0.41) | (0.025-0.060) |
|  | BLK | 7 | 1.5 | 0.27 |  | 0.45 | 0.078 |  | 2.0 | 0.31 |  | 0.51 | 0.083 |
|  |  |  | (0.18-4) | (0.088-0.47) |  | (0.18-1.0) | (0.041-0.13) |  | (0.18-4.0) | (0.088-0.47) |  | (0.18-1.0) | (0.041-0.13) |
|  | CWG | 7 | 2.1 | 0.45 |  | 0.48 | **0.10** |  | 2.9 | 0.53 |  | 0.61 | **0.11** |
|  |  |  | (0.63-5.8) | (0.15-1.0) |  | (0.19-1.4) | (0.046-0.21) |  | (0.63-5.8) | (0.15-1.0) |  | (0.19-1.4) | (0.046-0.21) |
|  | All suburban | 21 | 1.3 | **0.27** |  | 0.35* | **0.071*** |  | 2.1 | 0.36 |  | 0.45* | **0.081*** |
|  |  |  | (0.057-5.8) | (0.021-1.0) |  | (0.12-1.4) | (0.025-0.21) |  | (0.057-5.8) | (0.021-1.0) |  | (0.12-1.4) | (0.025-0.21) |
| Rural | GNA | 4 | 0.34* | 0.12* |  | 0.12* | 0.043* |  | 0.36* | 0.14* |  | 0.12* | 0.046* |
|  |  |  | (0.20-0.48) | (0.052-0.23) |  | (0.08-0.16) | (0.028-0.079) |  | (0.20-0.48) | (0.052-0.23) |  | (0.080-0.16) | (0.028-0.079) |
|  | ICY | 3 | 0.38^†^ | 0.13^†^ |  | 0.11* | 0.046* |  | 0.33* | 0.16* |  | 0.12* | 0.060* |
|  |  |  | (0.24-0.44) | (0.048-0.22) |  | (0.080-0.16) | (0.016-0.11) |  | (0.24-0.44) | (0.048-0.22) |  | (0.080-0.16) | (0.016-0.11) |
|  | LIR | 6 | 0.34* | 0.068* |  | 0.19 | 0.037 |  | 0.63* | 0.076* |  | 0.31 | 0.050 |
|  |  |  | (0.11-2.1) | (0.033-0.13) |  | (0.091-1.1) | (0.010-0.14) |  | (0.11-2.1) | (0.033-0.13) |  | (0.091-1.1) | (0.010-0.14) |
|  | All rural | 13 | 0.33* | 0.094* |  | 0.14* | 0.041* |  | 0.48* | 0.11* |  | 0.21* | 0.051* |
|  |  |  | (0.11-2.1) | (0.033-0.23) |  | (0.080-1.1) | (0.010-0.14) |  | (0.11-2.1) | (0.033-0.23) |  | (0.080-1.1) | (0.010-0.14) |

*****Mean and associated range include at least one less than the lower limit of quantitation (<LOQ) replacement value randomly generated from the range of <LOQs for the relevant contaminant category.

^†^Mean and associated range generated from <LOQ replacement values randomly generated from the range of <LOQs for CHLDs at ICY latrine. No CHLDs were detected above the LOQ at this latrine.

**Table S4.** Comparison of linear models assessing the effects of development zone (DevZone) or latrine site, δ^15^N, and scat lipid content (natural log-transformed percent lipid) on ∑_40_PCB, ∑_11_PBDE, ∑_6_DDT, and ∑_42_PAH concentrations in river otter (*Lontra canadensis*) scat samples. Akaike Information Criterion corrected for small sample size (AICc) and Akaike weights were used to select the best fit and most parsimonious model, noted in bold text. Simpler models were selected as most parsimonious if they had a ΔAICc less than or equal to two.

| Chemical Class | Model | AICc | ΔAICc | Adjusted R^2^ | Akaike weights |
| --- | --- | --- | --- | --- | --- |
| ∑_40_PCBs | **DevZone + ln(% lipid) + δ^15^N** | **154.9** | **0.0** | **0.852** | **6.9E-01** |
|  | DevZone + δ^15^N | 158.7 | 3.8 | 0.840 | 1.0E-01 |
|  | DevZone + ln(% lipid) | 158.9 | 4.0 | 0.840 | 9.4E-02 |
|  | DevZone + ln(% lipid) + (DevZone x ln(% lipid)) | 159.1 | 4.3 | 0.845 | 8.2E-02 |
|  | DevZone + δ^15^N + (DevZone x δ^15^N) | 161.4 | 6.5 | 0.840 | 2.7E-02 |
|  | Latrine site + ln(% lipid) | 167.4 | 12.5 | 0.853 | 1.3E-03 |
|  | Latrine site + ln(% lipid) + δ^15^N | 168.3 | 13.4 | 0.855 | 8.6E-04 |
|  | Latrine site + ln(% lipid) + (Latrine site x ln(% lipid)) | 173.7 | 18.8 | 0.895 | 5.7E-05 |
|  | Latrine site + δ^15^N | 174.5 | 19.6 | 0.837 | 3.9E-05 |
|  | DevZone | 181.1 | 26.3 | 0.774 | 1.4E-06 |
|  | Latrine site + δ^15^N + (Latrine site x δ^15^N) | 185.1 | 30.2 | 0.876 | 1.9E-07 |
|  | Latrine site | 191.6 | 36.7 | 0.785 | 7.5E-09 |
|  | δ^15^N | 214.0 | 59.1 | 0.630 | 1.0E-13 |
|  | ln(% lipid) + δ^15^N | 215.9 | 61.0 | 0.627 | 3.9E-14 |
|  | ln(% lipid) | 251.9 | 97.0 | 0.360 | 5.9E-22 |
| ∑_11_PBDE | DevZone + ln(% lipid) + (DevZone x ln(% lipid)) | 163.7 | 0.0 | 0.736 | 4.2E-01 |
|  | DevZone + ln(% lipid) + δ^15^N | 164.4 | 0.7 | 0.728 | 3.0E-01 |
|  | **DevZone + ln(% lipid)** | **164.6** | **0.9** | **0.722** | **2.7E-01** |
|  | DevZone + δ^15^N | 172.9 | 9.2 | 0.686 | 4.3E-03 |
|  | DevZone + δ^15^N + (DevZone x δ^15^N) | 173.4 | 9.6 | 0.696 | 3.4E-03 |
|  | Latrine site + ln(% lipid) | 175.2 | 11.5 | 0.737 | 1.3E-03 |
|  | Latrine site + ln(% lipid) + δ^15^N | 176.6 | 12.9 | 0.739 | 6.7E-04 |
|  | Latrine site + δ^15^N | 184.7 | 21.0 | 0.698 | 1.1E-05 |
|  | Latrine site + ln(% lipid) + (Latrine site x ln(% lipid)) | 188.8 | 25.1 | 0.791 | 1.5E-06 |
|  | DevZone | 190.5 | 26.8 | 0.587 | 6.3E-07 |
|  | ln(% lipid) + δ^15^N | 194.7 | 30.9 | 0.561 | 8.0E-08 |
|  | δ^15^N | 195.4 | 31.7 | 0.549 | 5.4E-08 |
|  | Latrine site + δ^15^N + (Latrine site x δ^15^N) | 199.4 | 35.7 | 0.756 | 7.5E-09 |
|  | Latrine site | 201.9 | 38.2 | 0.602 | 2.1E-09 |
|  | ln(% lipid) | 215.6 | 51.8 | 0.396 | 2.3E-12 |

| Chemicals Class | Model | AICc | ΔAICc | Adjusted R^2^ | Akaike weights |
| --- | --- | --- | --- | --- | --- |
| ∑_6_DDTs | **DevZone + ln(% lipid) + (DevZone x ln(% lipid))** | **193.5** | **0.0** | **0.653** | **5.3E-01** |
|  | DevZone + ln(% lipid) + δ^15^N | 194.8 | 1.3 | 0.640 | 2.9E-01 |
|  | DevZone + ln(% lipid) | 196.2 | 2.7 | 0.625 | 1.4E-01 |
|  | DevZone + δ^15^N + (DevZone x δ^15^N) | 199.7 | 6.2 | 0.621 | 2.5E-02 |
|  | ln(% lipid) + δ^15^N | 202.5 | 8.9 | 0.582 | 6.1E-03 |
|  | DevZone + δ^15^N | 202.9 | 9.4 | 0.586 | 4.8E-03 |
|  | Latrine site + ln(% lipid) + δ^15^N | 204.3 | 10.7 | 0.668 | 2.5E-03 |
|  | δ^15^N | 206.1 | 12.5 | 0.650 | 1.0E-03 |
|  | Latrine site + ln(% lipid) | 206.3 | 12.8 | 0.550 | 8.8E-04 |
|  | Latrine site + δ^15^N | 210.8 | 17.2 | 0.625 | 9.8E-05 |
|  | Latrine site + ln(% lipid) + (Latrine site x ln(% lipid)) | 214.9 | 21.4 | 0.740 | 1.2E-05 |
|  | ln(% lipid) | 220.5 | 26.9 | 0.447 | 7.5E-07 |
|  | DevZone | 223.6 | 30.1 | 0.431 | 1.6E-07 |
|  | Latrine site + δ^15^N + (Latrine site x δ^15^N) | 227.5 | 33.9 | 0.688 | 2.3E-08 |
|  | Latrine site | 235.2 | 41.6 | 0.450 | 4.9E-10 |
| ∑_37_PAH | DevZone + ln(% lipid) + δ^15^N | 224.4 | 0.0 | 0.577 | 4.6E-01 |
|  | **DevZone + δ^15^N** | **225.7** | **1.3** | **0.561** | **2.5E-01** |
|  | DevZone + ln(% lipid) | 225.9 | 1.5 | 0.559 | 2.2E-01 |
|  | DevZone + ln(% lipid) + (DevZone x ln(% lipid)) | 230.0 | 5.6 | 0.550 | 2.8E-02 |
|  | DevZone + δ^15^N + (DevZone x δ^15^N) | 230.4 | 6.0 | 0.547 | 2.3E-02 |
|  | δ^15^N | 231.6 | 7.2 | 0.503 | 1.3E-02 |
|  | ln(% lipid) + δ^15^N | 232.3 | 7.9 | 0.507 | 8.8E-03 |
|  | DevZone | 239.5 | 15.1 | 0.453 | 2.4E-04 |
|  | Latrine site + ln(% lipid) | 242.8 | 18.4 | 0.544 | 4.7E-05 |
|  | Latrine site + ln(% lipid) + δ^15^N | 244.4 | 20.0 | 0.546 | 2.1E-05 |
|  | Latrine site + δ^15^N | 246.8 | 22.4 | 0.517 | 6.5E-06 |
|  | ln(% lipid) | 251.4 | 27.0 | 0.338 | 6.3E-07 |
|  | Latrine site | 257.6 | 33.2 | 0.419 | 2.9E-08 |
|  | Latrine site + ln(% lipid) + (Latrine site x ln(% lipid)) | 262.7 | 38.3 | 0.602 | 2.2E-09 |
|  | Latrine site + δ^15^N + (Latrine site x δ^15^N) | 262.8 | 38.4 | 0.602 | 2.1E-09 |

**Table S5.** Predicted mean concentrations, standard errors (SE), and 95% confidence intervals (CI) for the best-fitting model for ∑_6_DDTs (ln values) using the mean lipid content (ln values) in each of three development zones (grand mean results presented in Table 2) along the Green-Duwamish River, Washington State, USA. Development zones are based on impervious surface land cover as defined in the methods. Pairwise comparisons were conducted using the Sidak adjustment for multiple comparisons. Groups with the same lower case letter were not significantly different from each other.

| **POP Class** | **Mean lipid content** | **Development Zone** | **Estimated Mean** | **SE** | **95% CI** |  |
| --- | --- | --- | --- | --- | --- | --- |
|  |  |  |  |  |  |  |
| ln∑_6_DDTs | Rural  ln(lipids) =  -0.17 | Industrial | 0.91 | 0.152 | 0.537 – 1.28 | c |
|  |  | Suburban | 0.654 | 0.263 | 0.0091 – 1.30 | b |
|  |  | Rural | -0.66 | 0.371 | -1.57 – 0.25 | a |
|  |  |  |  |  |  |  |
| ln∑_6_DDTs | Suburban  ln(lipids) =  -0.70 | Industrial | 0.595 | 0.183 | 0.146 – 1.04 | c |
|  |  | Suburban | -0.178 | 0.196 | -0.660 – 0.304 | b |
|  |  | Rural | -1.338 | 0.272 | -2.00 – -0.672 | a |
|  |  |  |  |  |  |  |
| ln∑_6_DDTs | Industrial  ln(lipids) =  -1.00 | Industrial | 0.416 | 0.221 | -1.25 – 0.957 | b |
|  |  | Suburban | -0.648 | 0.22 | -1.19 – -0.109 | b |
|  |  | Rural | -1.722 | 0.25 | -2.34 – -1.109 | a |
|  |  |  |  |  |  |  |

**Table S6.** Predicted mean concentrations, standard errors (SE), and 95% confidence intervals (CI) for percent lipid and δ^15^N in river otter (*Lontra canadensis*) scat samples from three development zones along the Green-Duwamish River in Washington State, USA. Development zones are based on impervious surface land cover as defined in the methods. Groups with the same lower case letter were not significantly different from each other.

|  | **Development Zone** | **Estimated Mean** | **SE** | **95% CI** | |  | |  |  |  |
| --- | --- | --- | --- | --- | --- | --- | --- | --- | --- | --- |
| ln(lipids) | Industrial | -0.174 | 0.127 | -0.485 – 0.138 | | b | |  |  |  |
|  | Suburban | -0.7 | 0.164 | -1.10 – -0.298 | | a | |  |  |  |
|  | Rural | -1.038 | 0.208 | -1.55 – 0.527 | | a | |  |  |  |
|  |  |  |  | |  | |  | |  |  |
| δ^15^N | Industrial | 11.01 | 0.508 | 9.76 – 12.3 | | c | |  |  |  |
|  | Suburban | 7.38 | 0.656 | 5.78 – 8.99 | | b | |  |  |  |
|  | Rural | 3.66 | 0.834 | 1.61 – 5.70 | | a | |  |  |  |

**Table S7.** Arithmetic means and ranges of ∑_11_PBDE concentrations (mg/kg lw) in river otter (*Lontra canadensis*) scat collected from 12 latrines in three development zones along the Green-Duwamish River in Washington State, USA, and their conversions to ∑_11_PBDE liver tissue concentration (mg/kg lw) estimates following La Guardia et al. (2020) body burden estimates. Bold values are scat samples that exceeded half the no-observed-adverse-effect-level (NOAEL; 1.19 mg/kg lw). River KM begins with 0 at the mouth of the Green-Duwamish River and increases moving upstream (see also Fig.1). Development zones are based on impervious surface land cover as defined in the methods.

| **Development Zone** | **Latrine** | **River KM** | **N** | **∑_11_PBDE** | **∑_11_PBDE range** | **∑_11_PBDE liver tissue** | **∑_11_PBDE liver tissue range** |
| --- | --- | --- | --- | --- | --- | --- | --- |
| Industrial | HIM | 0 | 3 | 0.32 | 0.071 – 0.80 | 0.17 | 0.038 – 0.43 |
|  | DIA | 1.1 | 2 | 1.3 | 0.42 – 2.2 | 0.71 | 0.23 – **1.2** |
|  | SPM | 5.5 | 12 | 0.50 | 0.27 – 0.97 | 0.27 | 0.15 – 0.52 |
|  | BOE | 6.3 | 2 | 0.62 | 0.60 – 0.64 | 0.34 | 0.032 – 0.35 |
|  | HAM | 7.2 | 8 | 0.49 | 0.086 – 1.0 | 0.26 | 0.046 – 0.54 |
|  | KCO | 7.4 | 8 | 0.73 | 0.074 – 1.2 | 0.39 | 0.040 – **0.65** |
|  | All industrial |  | 35 | 0.59 | 0.071 – 2.2 | 0.32 | 0.038 – 1.2 |
| Suburban | FGL | 14.5 | 7 | 0.45 | 0.1 – 1.0 | 0.24 | 0.054 – 0.54 |
|  | BLK | 16.1 | 7 | 0.50 | 0.18 – 0.87 | 0.27 | 0.097 – 0.47 |
|  | CWG | 32.2 | 7 | 0.37 | 0.078 – 1.3 | 0.20 | 0.042 – 0.70 |
|  | All suburban |  | 21 | 0.44 | 0.078 – 1.3 | 0.24 | 0.042 – 0.70 |
| Rural | GNA | 61.2 | 4 | 0.046 | 0.025 – 0.078 | 0.25 | 0.014 – 0.042 |
|  | ICY | 77.2 | 3 | 0.054 | 0.016 – 0.10 | 0.29 | 0.009 – 0.054 |
|  | LIR | 86.9 | 6 | 0.082 | 0.041 – 0.18 | 0.44 | 0.022 – 0.097 |
|  | All rural |  | 13 | 0.064 | 0.016 – 0.18 | 0.24 | 0.009 – 0.097 |

**Table S8.** Geometric mean concentrations and ranges of Σ_17_OCs in river otter (*Lontra canadensis*) scat collected from 12 latrines in three development zones along the Green-Duwamish River in Washington State, USA. This grouping is comparable to ΣOCs in Elliott et al. (2008), Guertin et al. (2010), and Huang et al. (2018). Concentrations are reported as ng/g wet weight (ww) and mg/kg lipid weight (lw). Maximum latrine and development zone contaminant concentrations are indicated in bold. Development zones are based on impervious surface land cover as defined in the methods. Σ_17_OCs = hexachloro-benzene (HCB); *alpha*-hexachlorocyclohexane, *beta*-hexachlorocyclohexane, *gamma*-hexachloro-cyclohexane (lindane); *cis*-chlordane, *trans*-chlordane, *cis*-nonachlor, *trans*-nonachlor, nonachlor III, heptachlor, heptachlor epoxide, oxychlordane; *p,p’*-DDD, *p,p’*-DDE, *p,p’*-DDT; dieldrin and mirex.

| **Development Zone** | **Latrine** | **River KM** | **N** | **Σ_17_OC** | |
| --- | --- | --- | --- | --- | --- |
|  |  |  |  | **ww** | **lw** |
| Industrial | HIM | 0 | 3 | 4.5 | 0.3 |
|  |  |  |  | (3.0-7.6) | (0.19-0.67) |
|  | DIA | 1.1 | 2 | 5.1 | 0.88 |
|  |  |  |  | (1.6-16) | (0.37-2.1) |
|  | SPM | 5.5 | 12 | 3.6 | 0.48 |
|  |  |  |  | (0.41-24) | (0.16-1.0) |
|  | BOE | 6.3 | 2 | 3.7 | 0.88 |
|  |  |  |  | (2.9-4.7) | (0.79-0.97) |
|  | HAM | 7.2 | 8 | 5.8 | 0.67 |
|  |  |  |  | (2.7-9.4) | (0.18-3.4) |
|  | KCO | 7.4 | 8 | **15** | **1.5** |
|  |  |  |  | (4.8-**45**) | (0.28-**6.3**) |
|  | All industrial |  | 35 | **5.8** | **0.69** |
|  |  |  |  | (0.41-**45**) | (0.16-**6.3**) |
| Suburban | FGL | 14.5 | 7 | 1.7 | 0.37 |
|  |  |  |  | (0.14-7.0) | (0.052-1.1) |
|  | BLK | 16.1 | 7 | 3.2 | 0.55 |
|  |  |  |  | (0.58-11) | (0.25-0.87) |
|  | CWG | 32.2 | 7 | 4.7 | 1.0 |
|  |  |  |  | (1.3-19) | (0.31-3.3) |
|  | All suburban |  | 21 | 2.9 | 0.59 |
|  |  |  |  | (0.14-19) | (0.52-3.3) |
| Rural | GNA | 61.2 | 4 | 0.31* | 0.11* |
|  |  |  |  | (0.16-0.43) | (0.038-0.31) |
|  | ICY | 77.2 | 3 | 0.24* | 0.099* |
|  |  |  |  | (0.16-0.34) | (0.068-0.13) |
|  | LIR | 86.9 | 6 | 0.70* | 0.14* |
|  |  |  |  | (0.091-7.8) | (0.037-0.35) |
|  | All rural |  | 13 | 0.43* | 0.12* |
|  |  |  |  | (0.091-7.8) | (0.037-0.35) |

*Means and associated ranges include at least one less than the lower limit of quantitation (<LOQ) replacement value randomly generated from the range of <LOQs for the relevant contaminant category.


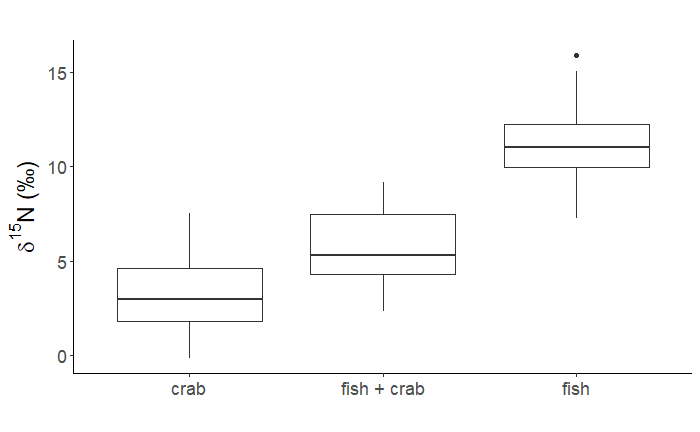


a

b

c

**Figure S1.** δ^15^N in river otter scat compared to gross field characterization of hard parts present in otter scat during sample collection along the Green-Duwamish River, WA. Scat were characterized as containing either fish hard parts (fish), crustacean hard parts (crab), or an obvious mixture of the two (fish + crab). The patterns of δ^15^N in river otter scat were representative of the categorical field characterizations of prey hard parts visible during scat collection and aligned with the general trophic levels associated with these prey items. Sidak multiple comparisons test identified significant differences in δ^15^N across all diet categories (*p* < 0.05 for all comparisons), denoted by different lower-case letters.

**Genetics details**

Methods

Swabs stored in 1 x lysis buffer (Rutledge et al., 2009) were extracted with a Qiagen DNAeasy tissue extraction kit following manufacturer’s instructions (Qiagen). Briefly, 25 µl of proteinase K (Qiagen) was added to each sample and the samples were incubated at 65°C for 2 hours. Subsequently, a second spike with 25 µl of proteinase K was added and samples were incubated at 37°C for 12 hours. Then, an equal volume of AL buffer was added to each sample and the samples were incubated at 65°C for 10 minutes. Afterwards, 500 µl of 95% ethanol was added. Each sample was then loaded into a silica column and centrifuged to bind DNA to the membrane. Each column was then washed first with 500 µl of AW1 buffer followed by 500 µl of AW2 buffer. Finally, DNA was eluted using 50 mL of TE 0.1 buffer (10 mM Tris-HCl, 0.1 mM EDTA, pH 8.0), heated to 65°C, and spun for 1 minute at 3500×g in a microcentrifuge. Extracted DNA was quantified with a NanoDrop 8000.

Extracted DNA samples were amplified at 14 microsatellite loci (Table S9). PCR reactions were carried out in 7 μl volumes and contained: 1x PCR buffer, 2.0 mM MgCl_2_, 0.6 μg/ml of BSA, 0.2 - 0.4 μM of each primer pair, 0.2 μM of each dinucleotide triphosphate, 0.5 unit of *Taq* polymerase (Invitrogen Life Technologies), and ~5 ng of DNA template. The thermocycling protocol consisted of a denaturation step at 95°C for 10 min, 30 cycles of 94°C for 30s, an annealing step for 60s at 55°C - 59°C depending on multiplex, and an extension at 72°C for 1 min. A final extension time of 65°C for 15 min was added to completely amplify amplicons. An ABI3700 genetic analyzer was used to separate fragments. For allele scoring, the program GENEMARKER v. 1.9.1 (SoftGenetics LLC, State College, Pennsylvania, USA) was used. A negative control was included to check for contamination and two positive controls were included to standardize allele scoring on each PCR plate. Re-amplification was employed to check ambiguous alleles (e.g., unusual morphologies) and alleles with low relative fluorescence units heights.

We tested for potential genotyping errors, null alleles, and allelic dropouts using the software Micro-Checker v. 2.2.3 (van Oosterhout et al., 2004). We identified identical genotypes/individuals with the program Allelematch version 2.5 (Galpern et al. 2012).

We tested for significant deviations from Hardy-Weinberg equilibrium and linkage disequilibrium with the program Genepop v. 4.2 (Rousset, 2008). Common summary statistics for genetic diversity like observed and expected heterozygosity were calculated with GenAlEx 6.5 (Peakall & Smouse, 2012). Pairwise relatedness was assessed with the program ML-RELATE (Kalinowski, 2006).

Results

There were no signs of large allele dropouts, allele scoring issues due to stutter bands, and null alleles detected in the dataset with Micro-Checker. For linkage disequilibrium tests, none of the 91 pairwise comparisons were significant after Bonferroni correction (7/91 were significant at the 0.05 level). In addition, none of the tests for deviations from Hardy-Weinberg equilibrium were significant after Bonferroni correction (2/14 were significant at the 0.05 level) and the F_IS_ values did not indicate an inbreeding pattern (Table S9).

**Table S9.** Number of alleles (NA), allelic range (A_R_), observed heterozygosity (H_O_), expected (H_E_), and F_IS_ plus p-values for microsatellite loci amplified from 18 unique river otters collected along the Green-Duwamish River, WA, USA.

| **Locus** | **N_A_** | **A_R_** | **Ho** | **He** | **F_IS_** | **P** |
| --- | --- | --- | --- | --- | --- | --- |
| **RIO01R** | 5 | 140-164 | 0.778 | 0.733 | -0.033 | 0.51 |
| **RIO02R** | 3 | 112-132 | 0.278 | 0.245 | -0.104 | 1.00 |
| **RIO04** | 6 | 98-116 | 0.333 | 0.458 | 0.299 | 0.04 |
| **RIO06R** | 5 | 126-142 | 0.889 | 0.725 | -0.198 | 0.97 |
| **RIO07R** | 6 | 90-100 | 0.889 | 0.750 | -0.157 | 0.92 |
| **RIO08** | 3 | 204-212 | 0.222 | 0.202 | -0.071 | 1.00 |
| **RIO09** | 2 | 251-253 | 0.889 | 0.494 | -0.790 | 1.00 |
| **RIO11** | 4 | 155-161 | 0.722 | 0.735 | 0.045 | 0.32 |
| **RIO12** | 3 | 207-211 | 0.389 | 0.508 | 0.261 | 0.19 |
| **RIO17** | 2 | 170-174 | 0.222 | 0.278 | 0.227 | 0.39 |
| **RIO18** | 10 | 138-162 | 0.556 | 0.773 | 0.308 | 0.01 |
| **RIO03** | 3 | 194-218 | 0.125 | 0.119 | -0.017 | 1.00 |
| **RIO10** | 7 | 220-253 | 0.765 | 0.813 | 0.090 | 0.18 |
| **RIO15R** | 2 | 141-143 | 0.167 | 0.239 | 0.329 | 0.27 |

A matrix of relatedness revealed that of the 153 possible dyadic comparisons, 83% were unrelated (Table S10). There were four full-sibling, 17 half-sibling, and five parent-offspring relationships. Among all 26 related pairs, nine (35%) deposited scat at the same latrine; another 12 (46%) pairs deposited scat within the same geographic zone. The farthest apart that related individuals were detected was 55 km (female half-siblings, GNA to SPM; Fig 1). This indicated that closely related individuals are usually found in close geographic proximity as expected in otters, a species that at least for some time each year forms family groups.

**Table S10.** A matrix of relatedness for 18 unique river otters identified from microsatellite DNA analysis of scat collected along the Green-Duwamish River, WA. The three letter code (e.g., CWG) represents the latrine from which the scat was collected (see Fig. 1). U = unrelated; HS = half-sibling; FS = full-sibling; PO = parent-offspring.

In a previous study, higher amplification success rates were recorded for the protocol applied here (Klütsch & Thomas, 2018). Klütsch & Thomas (2018) collected scats during winter in a region where temperatures ranged from -11 to 46ºF and scat was commonly frozen overnight, while in the current study, temperature ranged from 54.5 to 65.6ºF. This suggests that temperature may have played a role in our reduced amplification rates, as has been shown by others (Hájková et al., 2006; Klütsch & Thomas, 2018). An interannual comparison of sample meta-data partially supports this idea. For 2016, mean temperature during scat collection was significantly lower for the scat samples that amplified than for those that did not (60.6 vs 65.6ºF, t=2.9, p<0.006). In 2017, scat was swabbed consistently before sunrise to potentially increase DNA yield. Although average temperature of all 2017 samples was significantly lower than 2016 (55.3 vs 64.7ºF, t=10.0, p<0.0001) and significantly below the mean of 2016 successfully amplified samples (55.3 vs 60.6ºF, t=3.6, p=0.0005), amplification success rates remained low (18% vs 17% in 2016). Meanwhile, unlike in 2016, there were no differences in mean temperatures between samples that amplified and those that did not (54.5 vs 55.4ºF, t=0.6, p=0.5), suggesting that other factors also affected amplification success rates.

Samples that consisted of anal jellies or scat with notable amounts of anal jelly in them (here referred to collectively as anal jellies) amplified at a proportionately much higher rate than their percent in total samples. In 2016, anal jellies comprised 19% of all samples, but 47% of successful amplifications (χ^2^=50.9, p<0.0001); in 2017, anal jellies comprised 23% of all samples, but 46% of successful amplifications (χ^2^=29.9, p<0.0001). Other studies have also found amplification success rates to be higher when using anal jellies (Hájková et al., 2006; Lampa et al., 2008; Mowry et al., 2011; Lerone et al., 2014; Sittenthaler et al., 2021; see also Huang et al., 2018 and references therein). While the collection of anal jellies apparently leads to higher amplification success rates, some have found the potential for male sampling bias presumably due to sex differences in marking behavior (Lampa et al. 2015), though others have not (Dallas, et al., 2003; Kruuk, 1992). Careful consideration of this tradeoff is warranted depending on the goals of the study. Overall, the results suggest that various microclimatic conditions should be considered when planning otter population genetic studies and timing of sample collection periods.

References

Dallas, J. F., Coxon, K. E., Sykes, T., Chanin, P. R. F., Marshall, F., Carss, D. N., Bacon, P. J., Piertney, S. B., & Racey, P. A. (2003). Similar estimates of population genetic composition and sex ratio derived from carcasses and faeces of Eurasian otter (*Lutra lutra*). *Molecular Ecology, 12*, 275–282.

Galpern, P., Manseau, M., Hettinga, P., Smith, K., & Wilson, P. (2012). Allelematch: an R package for identifying unique multilocus genotypes where genotyping error and missing data may be present. *Molecular Ecology Resources, 12*(4), 771–778. https://doi.org/10.1111/j.1755-0998.2012.03137.x

Hájková, P., Zemanová, B., Bryja, J., Hájek, B., Roche, K., Tkadlec, E., & Zima, J. (2006). Factors affecting success of PCR amplification of microsatellite loci from otter faeces. *Molecular Ecology Notes, 6*, 559–562. https://doi.org/10.1111/j.1471-8286.2006.01269.x

Huang, A. C., Nelson, C., Elliott, J. E., Guertin, D. A., Ritland, C., Drouillard, K., Cheng, K. M., & Schwantje, H. M. (2018). River otters (*Lontra canadensis*) "trapped" in a coastal environment contaminated with persistent organic pollutants: Demographic and physiological consequences. *Environmental Pollution, 238*, 306-316. https://doi.org/10.1016/j.envpol.2018.03.035.

Kalinowski, S. T., Wagner, A. P., & Taper, M. L. (2006). ml-relate: a computer program for maximum likelihood estimation of relatedness and relationship. *Molecular Ecology Notes, 6*, 576–579. https://doi.org/10.1111/j.1471-8286.2006.01256.x

Klütsch, C. F. C., & Thomas, P. J. (2018). Improved genotyping and sequencing success rates for North American river otter (*Lontra canadensis*). *European Journal of Wildlife Research, 64*, 16. https://doi.org/10.1007/s10344-018-1177-y

Kruuk, H. (1992). Scent marking by otters (*Lutra lutra*): Signalling the use of resources. *Behavorial Ecology, 3*, 133–140.

Lampa, S., Gruber, B., Henle, K., & Hoehn, M. (2008). An optimisation approach to increase DNA amplification success of otter faeces. *Conservation Genetics, 9*, 201–210.

Lampa, S., Mihoub, J. B., Gruber, B., Klenke, R., & Henle, K. (2015). Noninvasive genetic mark-recapture as a means to study population sizes and marking behaviour of the elusive Eurasian Otter (*Lutra lutra*). *PloS One, 10*(5), e0125684. https://doi.org/10.1371/journal.pone.0125684

Lerone, L., Mengoni, C., Carpaneto, G. M., Randi, E., & Loy, A. (2014). Procedures to genotype problematic non-invasive otter (*Lutra lutra*) samples. *Acta Theriologica 59*, 511–520.

Mowry, R. A., Gompper, M. E., Beringer, J., & Eggert, L. S. (2011). River otter population size estimation using noninvasive latrine surveys. *Journal of Wildlife Management, 75*, 1625–1636. https://doi.org/10.1002/jwmg.193

Peakall, R., & Smouse, P. E. (2012). GenAlEx 6.5: genetic analysis in Excel. Population genetic software for teaching and research – an update. *Bioinformatics, 28*(19), 2537–2539. https://doi.org/10.1093/bioinformatics/bts460

Rousset, F. (2008). Genepop'007: a complete reimplementation of the Genepop software for Windows and Linux. *Molecular Ecology Resources, 8*, 103–106.

Rutledge, L. Y., Holloway, J. J., Patterson, B. R., & White, B. N. (2009). An improved field method to obtain DNA for individual identification from scat. *The Journal of Wildlife Management 73*, 1430–1435.

Sittenthaler, M., Schöll, E. M., Leeb, C., Haring, E., Parz-Gollner, R., & Hackländer, K. (2021). Factors influencing genotyping success and genotyping error rate of Eurasian otter (*Lutra lutra*) faeces collected in temperate Central Europe. *European Journal of Wildlife Research, 67*, 2.

van Oosterhout, C., Hutchinson, W. F., Wills, D. P. M., & Shipley, P. (2004). micro-checker: software for identifying and correcting genotyping errors in microsatellite data. *Molecular Ecology Notes, 4*, 535-538. https://doi.org/10.1111/j.1471-8286.2004.00684.x
